# Out of the Qinghai-Tibetan plateau: Origin, evolution and historical biogeography of *Morchella* (both Elata and Esculenta clades)

**DOI:** 10.3389/fmicb.2022.1078663

**Published:** 2022-12-28

**Authors:** Qing Meng, Zhanling Xie, Hongyan Xu, Jing Guo, Yongpeng Tang, Ting Ma, Qingqing Peng, Bao Wang, Yujing Mao, Shangjin Yan, Jiabao Yang, Deyu Dong, Yingzhu Duan, Fan Zhang, Taizhen Gao

**Affiliations:** ^1^College of Ecological and Environment Engineering, Qinghai University, Xining, Qinghai, China; ^2^State Key Laboratory Breeding Base for Innovation and Utilization of Plateau Crop Germplasm, Qinghai University, Xining, Qinghai, China; ^3^Academy of Agriculture and Forestry Sciences, Qinghai University, Xining, Qinghai, China; ^4^State-owned Forest Farm of Tianjun County, Delingha, Qinghai, China; ^5^Forestry and Grassland Station of Tianjun County, Delingha, Qinghai, China

**Keywords:** *Morchella*, Qinghai-Tibet plateau subkingdoms, multigene phylogenetics, age estimation, phylogeographic structure

## Abstract

**Introduction:**

Morchella has become a research hotspot because of its wide distribution, delicious taste, and phenotypic plasticity. The Qinghai-Tibet Plateau subkingdoms (QTPs) are known as the cradle of Ice age biodiversity. However, the diversity of Morchella in the QTPs has been poorly investigated, especially in phylogenetic diversity, origin, and biogeography.

**Methods:**

The genealogical concordance phylogenetic species recognition (GCPSR, based on Bayesian evolutionary analysis using sequences from the internal transcribed spacer (ITS), nuclear large subunit rDNA (nrLSU), translation elongation factor 1-α (EF1-α), and the largest and second largest subunits of RNA polymerase II (RPB1 and RPB2)), differentiation time estimation, and ancestral region reconstruction were used to infer Morchella’s phylogenetic relationships and historical biogeography in the QTPs.

**Results:**

Firstly, a total of 18 Morchella phylogenetic species are recognized in the QTPs, including 10 Elata clades and 8 Esculenta clades of 216 individuals Secondly, the divergences of the 18 phylogenetic species were 50.24–4.20 Mya (Eocene-Pliocene), which was closely related to the geological activities in the QTPs. Furthermore, the ancestor of Morchella probably originated in the Northern regions (Qilian Shan, Elata cade) and southwestern regions (Shangri-La, Esculenta clade) of QTPs and might have migrated from North America (Rufobrunnea clade) via Beringian Land Bridge (BLB) and Long-Distance Dispersal (LDD) expansions during the Late Cretaceous. Moreover, as the cradle of species origin and diversity, the fungi species in the QTPs have spread out and diffused to Eurasia and South Africa starting in the Paleogene Period.

**Conclusion:**

This is the first report that Esculenta and Elata clade of Morchella originated from the QTPs because of orogenic, and rapid differentiation of fungi is strongly linked to geological uplift movement and refuge in marginal areas of the QTPs. Our findings contribute to increasing the diversity of Morchella and offer more evidence for the origin theory of the QTPs.

## Introduction

As a famous edible mushroom, *Morchella* owns important ecological functions and has high commercial value around the world (Dissanayake et al., 2021; Wu H. et al., 2021; Yu et al., 2022). It was popular research in taxonomy, species diversity, distribution, ecological diversity, phylogeny, biogeography, and artificial cultivation of *Morchella* species (Annette et al., 1978; Dahlstrom et al., 2000; Hao et al., 2019; Liu et al., 2019; Ali et al., 2021; Cao et al., 2022; Deng et al., 2022). The distribution of *Morchella* exhibits a high level of cryptic speciation and provincialism due to phenotypic plasticity and unreliable morphological species recognition (O’Donnell et al., 2011; Du et al., 2012a, 2015, 2018; Richard et al., 2015;). There are 72 phylogenetically distinct species in the world that have been recognized in this genus based on GCPSR (Loizides et al., 2015, 2016; Yatsiuk et al., 2016; Baroni et al., 2018; Du et al., 2019). In China, which was known as the center of *Morchella* species diversification and rich floristic diversity, a total of 16 Elata clades and 27 Esculenta clades phylospecies have been recorded (Du et al., 2012a,b, 2019). The phylogenetic species diversity of the *Morchella* in the Qinghai-Tibet Plateau subkingdom (QTPs) is not yet known, though.

Great changes in crustal movement on earth occurred during the Phanerozoic Paleozoic (4.6 billion years ago), after occurring of biological explosions and forming of thick sedimentary limestone (Mittermeier et al., 2011; Kate, 2022). The QTPs have been uplifted steadily and rhythmically since the end of the Early Tertiary period, which is famous as the “Third Pole” of the earth (Mao et al., 2021; Spicer et al., 2021; Xiong et al., 2022); and were known as the ecological barrier of China and even Asia based on complex topography, variable plateau climate, and rich ecosystem (Sun et al., 2012; Qin et al., 2015; Miehe et al., 2019; Liu et al., 2022). Several fossil records of QTPs illustrated the cradle of mammalian fauna and mountain flora in the Ice age (Deng et al., 2020; Mao et al., 2021; Wu Y. D. et al., 2021). For fungus in the QTPs, the opportunities for genetic variation and speciation were strongly increased *via* the isolation of high-altitude geographic and geological complexity. Furthermore, the QTPs are also called a refugium created by microclimatic variations that provided some protection, and situ speciation and relic persistence in the early originated lineages (Shrestha et al., 2010; Yuan, 2015; Yan et al., 2017; Phonepaserd et al., 2019; Mao et al., 2021). The rapidly radiational differentiation of *Morchella* species was reported in North America, Asia, and Europe. *M. rufobrunnea* (Rufobrunnea clade), as the oldest taxon of the genus *Morchella* and might diverge into the basal lineage, originated in western North America in the late Jurassic (O’Donnell et al., 2011; Du et al., 2012a, 2015; Loizides et al., 2016, 2021). During the emergence of the Mid-Continental Seaway and the subsequent uplift of the Rocky Mountains, the ancestors of the Esculenta and Elata clades spread to eastern North America from western North America in the early Cretaceous (Sanmartín et al., 2001; Donoghue, 2008; Du et al., 2015). After that, *Morchella* experienced widespread extinction due to the new folding of the Rocky Mountains and the uplift of the Sierra Madre Oriental Range in central North America. It is also speculated that the *Morchella* species spread to Europe and Asia from North America *via* the Thulean North Atlantic Land Bridge and the Beringian Land Bridge (Du et al., 2015). During the middle Miocene to the Pleistocene, the *Morchella* species in East Asia and Europe rapidly evolved under the gradually cooling climate and environmental heterogeneity caused by the rise of the Qinghai-Tibetan Plateau (O’Donnell et al., 2011; Du et al., 2012a, 2015). However, further information regarding the differentiation, speciation, origination, and evolution of *Morchella* in the QTPs is still unclear.

In this study, ITS rDNA sequences of 174 *Morchella* individuals collected from QTPs were generated for the aims of (i) investigating phylogenetic species diversity and geographic distribution of *Morchella* in the QTPs by using genealogical concordance phylogenetic species recognition (GCPSR); (ii) estimating divergence times of *Morchella* species lineages in the QTPs; (iii) defining the geographic distributions of ancestor lineages of the *Morchella* in the QTPs; and (iv) estimating divergence times and reconstructing ancestral regions for world-widely distributed species of *Morchella*.

## Materials and methods

### Sampling

**Figure 1:**
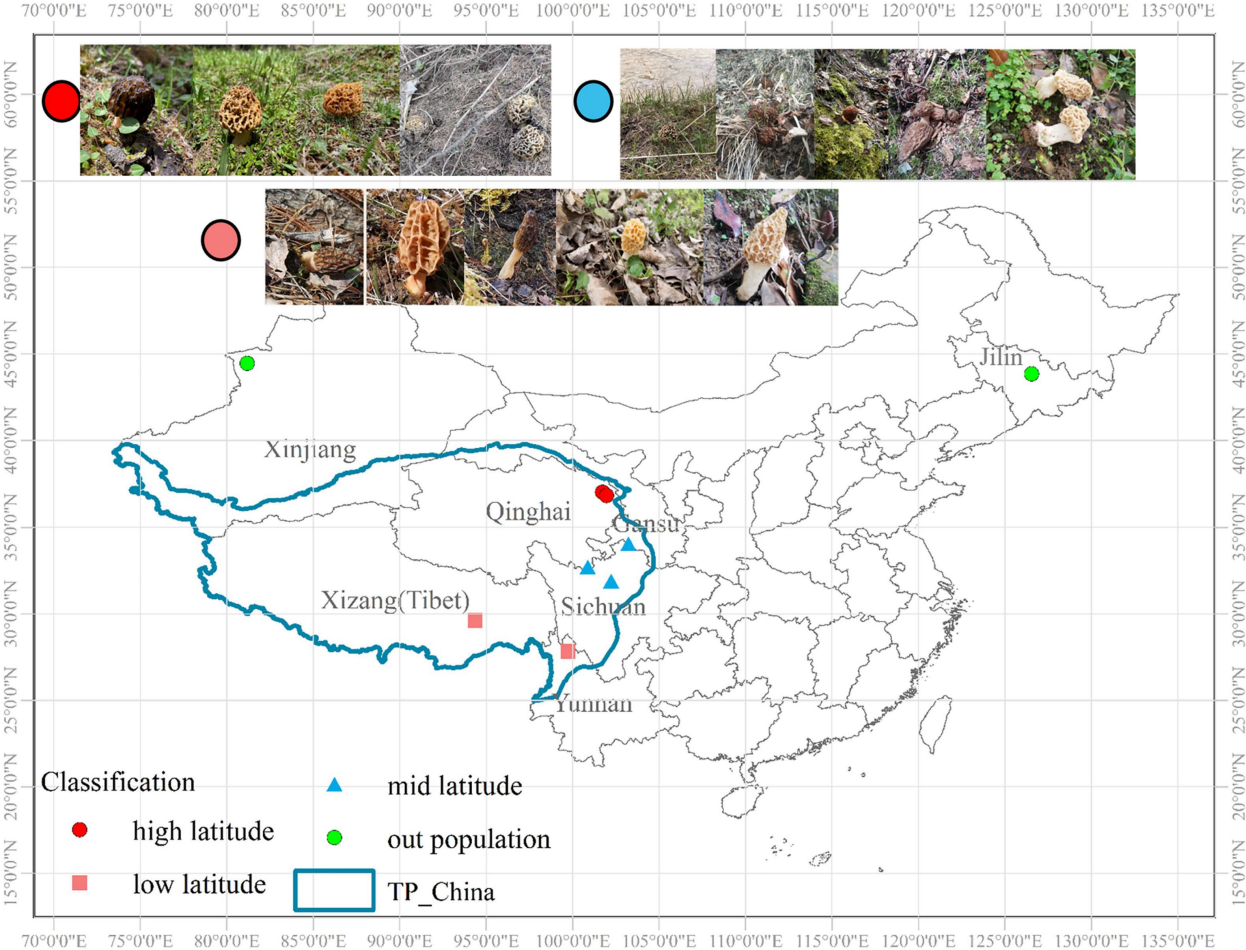
Geographical location and sampling site in Qinghai-Tibet Plateau subkingdom (QTPs) generated by ArcGIS v10.7. The red, pink, and blue sampling points are within the QTPs, where red is defined as the high-latitude region, blue is the middle-latitude region, and pink is the low-latitude region of the QTPs. The green sampling points are outside the QTPs.

A total of 216 individuals were collected from three parts, among which 174 individuals were from the QTPs and 30 individuals were from Xinjiang and 12 individuals were from Jilin University. Total 7 natural localities approximately extending the whole distribution range of QTPs, ranging from 26.07°N–44.01°N to 81.60°E–105.05°E during the harvesting seasons (April–June). We divided the sampling sites into the higher latitude (35°N–40°N), the lower latitude (25°N–30°N), and the middle latitude (30°N–35°N) regions according to the north latitude lines (Figure 1). Of these, there are 56, 64, and 92 individuals were collected from high, low, and middle latitudes of the northeastern, central-eastern, and southwestern in the QTPs, respectively. Micromorphological data were obtained from the dried specimens and observed under a light microscope following Baran and Boroń (2017). Voucher specimens were deposited in Extreme Environment Microbiology Laboratory, Qinghai University, Xining, China. The codes, locations, sampling year, and sample numbers of *Morchella* from the QTPs are shown in Supplementary Table S1.

### DNA extraction, PCR, and DNA sequencing

Total extracted DNAs of each sample were extracted using the modified 2× CTAB buffer method (Doyle and Doyle, 1987), checked by 1% agarose gel electrophoresis with ethidium bromide staining, and measured the concentration by spectrophotometer (Biospec-nano; Shimadzu). Five DNA gene fragments were analyzed, including those coding for RNA polymerase II largest subunit (*RPB1*) and second largest subunit (*RPB2*), translation elongation factor-1a (*TEF1*), along with two non-protein coding regions: internal transcribed spacer (ITS), nuclear large subunit rDNA (nrLSU). The PCR amplifications was performed according to Du et al. (2019), Baran and Boroń (2017), and Loizides et al. (2021). The primers used for PCR are listed in Supplementary Table S2. A total of 894 sequences of QTP *Morchella* were newly generated, including 216 ITS, 201 nrLSU, 161 *EF1-α*, 166 *RPB1*, and 125 *RPB2*. The ITS sequences generated in this study were combined with the representative 117 ITS sequences retrieved from GenBank (including 46 representatives of *Morchella* species recorded in NCBI) to identify the relationships between all of our individuals and the known related samples in GenBank. All newly generated sequences were submitted to GenBank (Supplementary Table S1).

### Sequence alignments and phylogenetic analyses

For phylogenetic analyses, the ITS gene datasets were analyzed *via* Maximum likelihood (ML) and Bayesian inference (BI): a 110-taxon, 864 bp Esculenta Clade data set; and (ii) a 101-taxon, 612 bp Elata Clade dataset. Five-gene datasets were analyzed *via* Maximum parsimony (MP), ML, and BI: (i) a 14-taxon, 3,380 bp Esculenta Clade data set; and (ii) a 24-taxon, 2,517 bp Elata Clade dataset. The genes extracted and aligned were using the MAFFT (version 7; Katoh and Standley, 2013). The conservative region was selected in Gblocks 0.91b and the vacancy gap in the data were treated as missing data. We performed MP, ML, and BI based on the combined sequences of five genes to reconstruct the relationships of *Morchella* and related taxa were subsequently conducted on PAUP∗ version 4.0 beta 10 (Swofford, 2002) and PholySuite v1.2.2 (Zhang et al., 2020). For the MP analysis was performed in PAUP∗ version 4.0 beta 10 (Swofford, 2002). All characters were equally weighted, and gaps were treated as missing data. Trees were inferred using the heuristic search option with TBR branch swapping and 1,000 random sequence additions. The PholySuite v1.2.2 contains programs for sequence alignment and phylogenetic analysis, such as ModelFinder to find the best model. For the ML analyses, all parameters were kept at their default settings, the concatenated dataset was partitioned into five parts by sequence region, and 1,000 Ml searches under the K80 (K2P) model with all model parameters estimated using the ModelFinder program, IQ-TREE v1.6.8 web server^1^ to carry out the ML searches. The MrBayes v3.2.6 for BI phylogenetic analyses also used ModelFinder to generate the best model (Ronquist et al., 2012; Du et al., 2012a; Zhu et al., 2019; Zhao et al., 2021). The phylogenetic trees were modified using FigTree v1.4.4 and the iTOL website.^2^

### Divergence dating analysis

we used BEAST v.2.6.6 (Chen et al., 2015; Zhu et al., 2019; Kim and Kim, 2022) to estimate the divergence times of *Morchella* phylospecies in the QTPs. In this study, we used *Floccularia luteovirens*, which is the endemic basidiomycete fungus in the QTPs, instead of *P. devonicus* as the calibration point 1 in Basidiomycota and Ascomycota (Taylor et al., 2004; Chen et al., 2015; Zhu et al., 2019; Guo et al., 2022). Normal distribution was applied by setting the mean and the standard deviation to 582.5 and 50.15, respectively. Calibration points 2 for analysis were obtained by including sequences of the following two species: *Verpa* and *M. rufobrunnea* (O’Donnell et al., 2011; Du et al., 2012a, 2015; Loizides et al., 2016, 2021). The origin time of *Morchella* was estimated in BEAST v.2.6.6 (Drummond and Rambaut, 2007) with the molecular clock and substitution models unlinked but with the trees linked for each gene partition. Two nuclear ribosomal RNA genes (ITS and nrLSU) and three protein-coding genes (*EF1-α*, *RPB1*, and *RPB2*), were concatenated for molecular dating. PholySuite v1.2.2 was also used to select the best models of evolution using the hierarchical likelihood ratio test. The GTR + I + G model was used for the *EF1-α* + *RPB1* + *RPB2* and the HKY + I + G model for the ITS + nrLSU data, based on the results from the PholySuite v1.2.2. The uncorrelated lognormal relaxed molecular clock and the Yule speciation prior set were used to estimate the divergence time and the corresponding credibility intervals by BEAUti 2. The Markov chain Monte Carlo (MCMC) analysis was 100 million generations, sampling parameters for every 1,000 generations. After discarding the first 10,000 (10%) trees as burn-in, the samples were summarized in a maximum clade credibility tree in TreeAnnotator v2.6.6 using a PP limit of 0.50 and summarizing the mean node heights. The means and 95% higher posterior densities (HPDs) of age estimates were obtained from the combined outputs using Tracer. FigTree v1.4.2 and iTOL website^3^ was used to visualize the resulting tree and to obtain the means and 95% HPD. A 95% HPD marks the shortest interval that contains 95% of the values sampled.

### Biogeographic analysis

Ancestral area reconstruction and estimating spatial patterns of geographic diversification within *Morchella* in the QTPs were inferred using the Bayesian binary method (BBM) and statistical dispersal-vicariance analysis (S-DIVA) as implemented in Reconstruct Ancestral State in Phylogenies (RASP v3.1). The distribution range of the Elata clade in the QTPs was divided into five regions, consisting of A (Qinghai), B (Tibet), C (Gansu), D (Xinjiang), and E (Other). And the distribution range of the Esculenta clade in the QTPs was also divided into five regions, consisting of (A) Qinghai, (B) Gansu, (C) Sichuan, (D) Yunnan, and E (Other). For the BBM analysis, we used all post-burn-in trees obtained from the BEAST v2.6.6 analysis. The BBM was run using the fixed state frequencies model (Jukes-Cantor) with equal among-site rate variation for 50,000 generations, 10 chains each, and two parallel runs. In statistical dispersal-vicariance analysis (S-DIVA), the frequencies of an ancestral range at a node in ancestral reconstructions are averaged over all trees. In addition, the world-widely distribution of 8 *Morchella* species (Supplementary Table S2), *M. spongiola*, *M. esculenta*, *M. crassipes*, *M. eohespera*, *M. eximia*, *M. costata*, *Mel*-13, *Mel*-14, were downloaded in NCBI to estimate the differentiation time and reconstruction ancestral area using BEAST v2.6.6 and RASP v3.1, respectively. ArcGIS v10.7 was used to visualize the geographic distribution and possible dispersal routes of *Morchella* (Yu et al., 2015; Kim et al., 2019; Kim and Kim, 2022).

## Results

### The recognition of 18 phylospecies of *Morchella* in the QTPs

**Figure 2:**
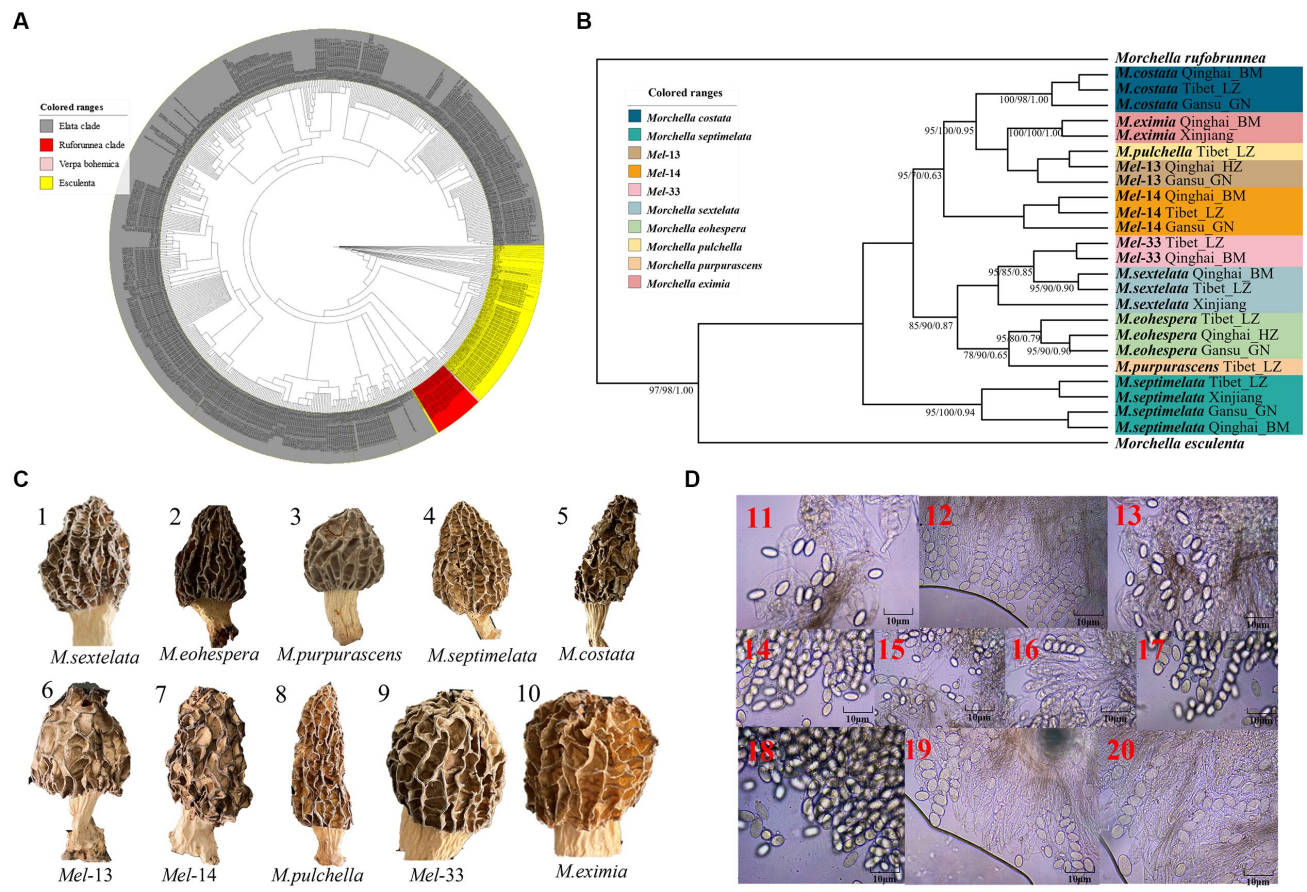
Species recognition of the *Morchella* in Elata clade from the QTPs. **(A)** Bayesian inference phylogenetic analyses of the Elata clade were inferred from 115 internal transcribed spacer (ITS) sequences representing a total of 10 phylospecies. **(B)** Phylogenetic analyses of the Elata clade were inferred from 120 (24*5) multi-genes (ITS+LSU + *EF1-α + RPB1 + RPB2*) sequences representing a total of ten phylospecies. Branches are labeled where MP/ML support is greater than 60% and collapsed below that support threshold. BPP is labeled were greater than 0.95. **(C)** Morphological diversity of the 10 Elata clades’ ascocarps from the QTPs: *M. sextelata/Mel-*6 (1), *M. norvegiensis* = *M. eohespera/Mel-*19 (2), *M. purpurascens/Mel-*20 (3), *M. septimelata/Mel-*7 (4), *M. costata* (5), *M. deliciosa/Mel*-13 (6), *Mel*-14 (7), *M. pulchella/Mel*-31 (8), *Mel*-33 (9), *M. eximia/Mel*-5 (10). **(D)** Micromorphological ascospores of the 10 Elata clades.

**Figure 3:**
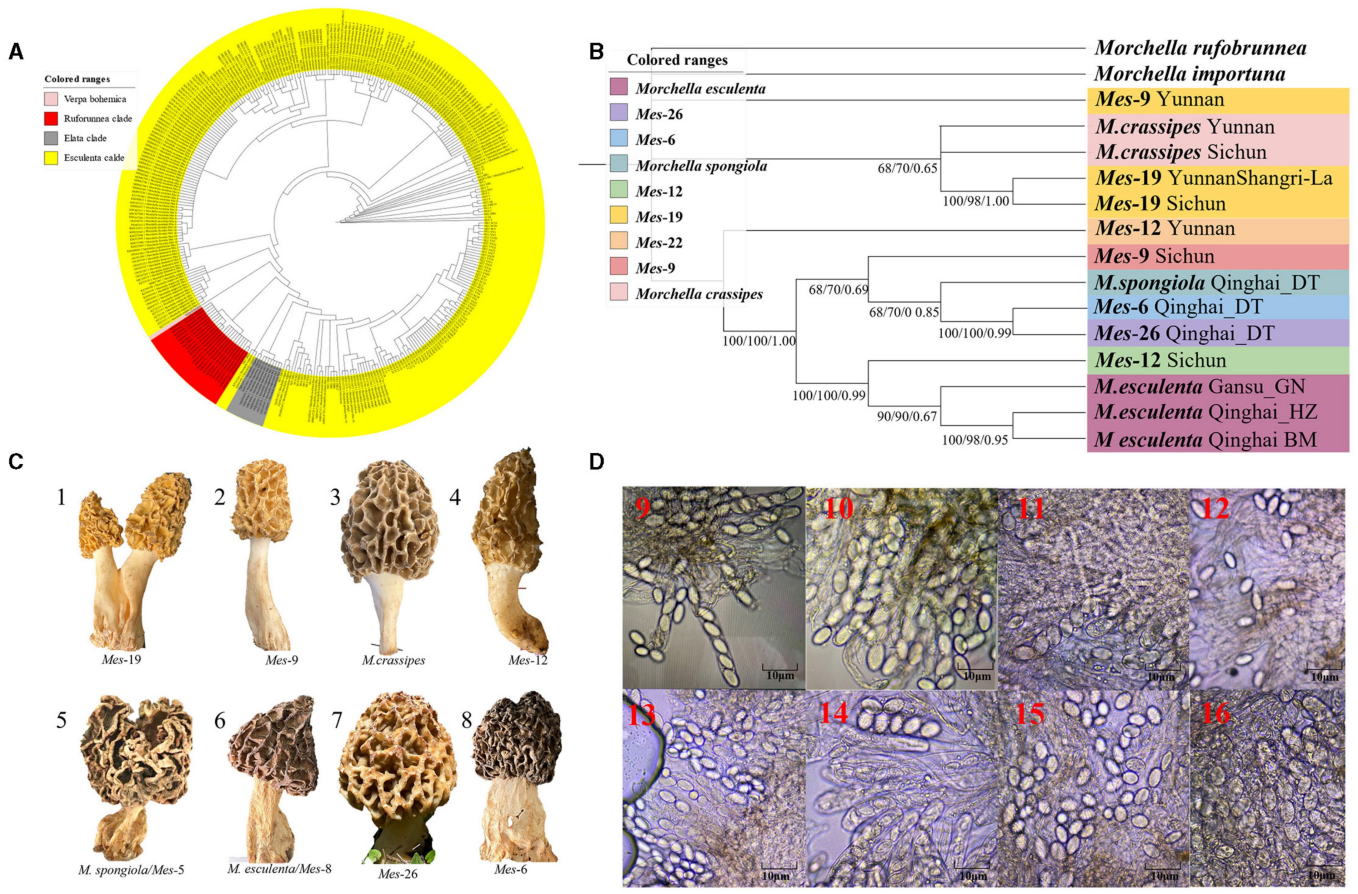
Species recognition of the *Morchella* in Esculenta clade from the QTPs. **(A)** Bayesian inference phylogenetic analyses of the Elata clade were inferred from 101 ITS sequences representing a total of 8 phylospecies. **(B)** Phylogenetic analyses of the Esculenta clade were inferred from 70 (14*5) multi-genes (ITS+LSU + *EF1-α + RPB1 + RPB2*) sequences representing a total of 8 phylospecies. Branches are labeled where MP/ML support is greater than 60% and collapsed below that support threshold. BPP is labeled were greater than 0.95. **(C)** Morphological diversity of the 8 Esculenta clades’ ascocarps from the QTPs: *Mes*-19 (1); *Mes*-9 (2), *M. crassipes* (3), *Mes*-12 (4), *M. vulgaris* = *M. spongiola/Mes*-5 (5), *M. esculenta/Mes*-8 (6), *Mes*-26 (7), *Mes*-6 (8). **(D)** Micromorphological ascospores of the 8 Escuenlta clades.

A total of 216 individuals of Morchella were classified into Esculenta and Elata clades, there is no Rufobrunnea clade. A total of 101 individuals clustered with 10 phylogenetic species, including *Mel*-14, *M. deliciosa*/*Mel*-13, *M. norvegiensis* = *M. eohespera/Mel*-19, *Morchella eximia/Mel*-5, *Morchella costata, Morchella sextelata/Mel*-6, *Morchella septimelata/Mel*-7, *Morchella purpurascens/Mel-20, Mel*-33, and *Morchella pulchella/Mel*-31 belongs to Elata clade (Figures 2A–C); and a total of 101 individuals clustered with 8 phylogenetic species, including *M. vulgaris* = *M. spongiola/Mes*-5, *Mes*-9, *Mes*-12, *Mes*-26, *Morchella crassipes, Morchella esculenta/Mes*-8, *Mes*-19, and *Mes*-6 belongs to Esculenta clade (Figures 3A–C). All *Morchella* species also exhibit extreme bradytelic morphological evolution as evidenced by the retention of the ancestral ascocarp body plan (Figures 2D, 3D). Overall, we identified 18 phylospecies that was widely distributed in the QTPs.

### The estimation of the divergence time of *Morchella* in the QTPs

**Figure 4:**
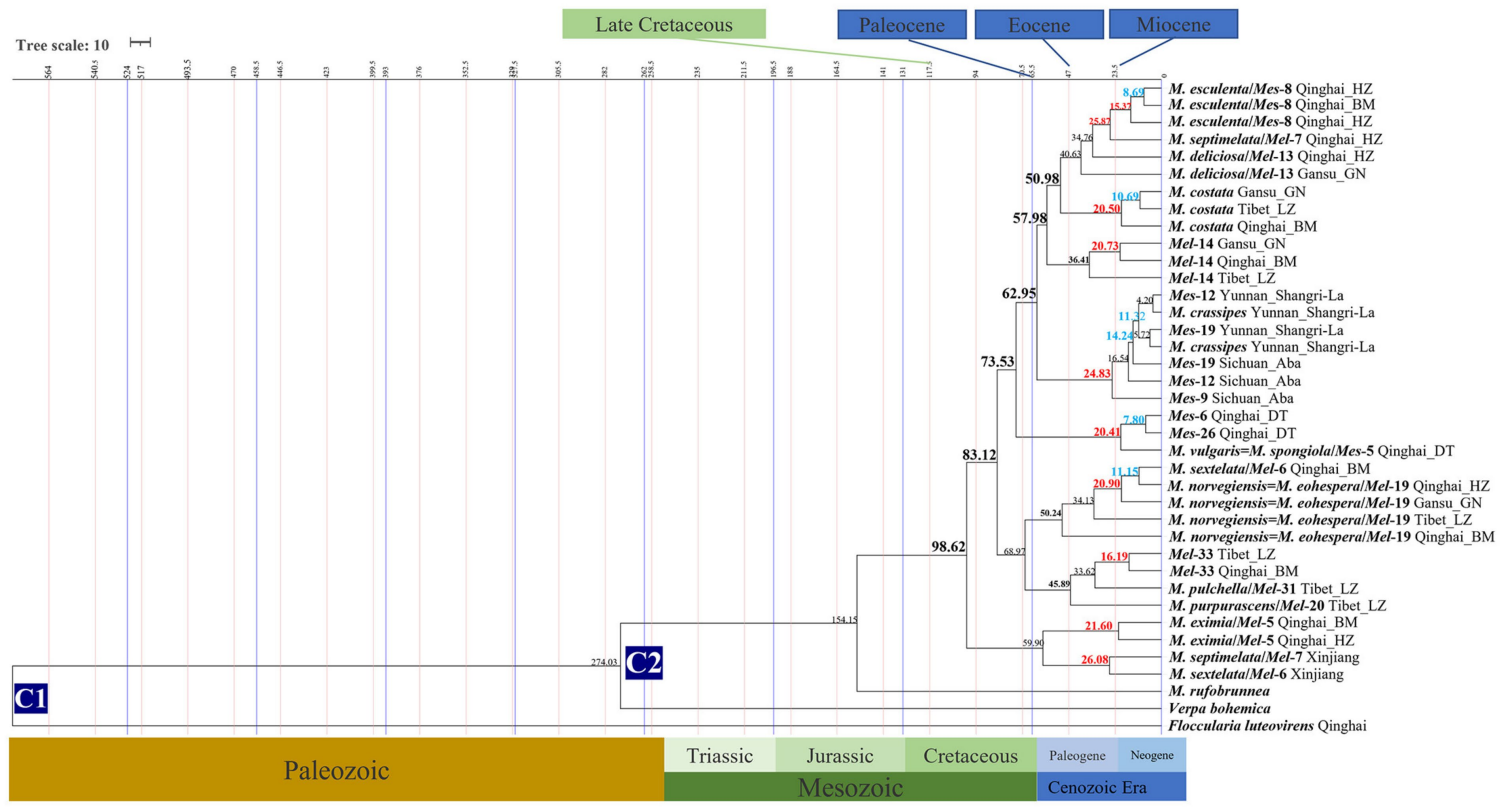
Chronogram and estimated divergence times of *Morchella* in QTPs generated by molecular clock analysis using the two concatenated datasets (ITS + LSU and  *EF1-α + RPB1 + RPB2*) dataset. The chronogram was obtained using the Ascomycota-Basidiomycota divergence time of 582.08 Mya as the calibration point 1. The *Morchella*-*Verpa bohenica* divergence time of 274.06 Mya as the calibration point 2. The calibration point and objects of this study are marked in the chronogram. The geological time scale is millions of years ago (Mya). The red font is defined as the first uplift of the QTPs, and the blue font is defined as the second uplift of the QTPs.

**Figure 5:**
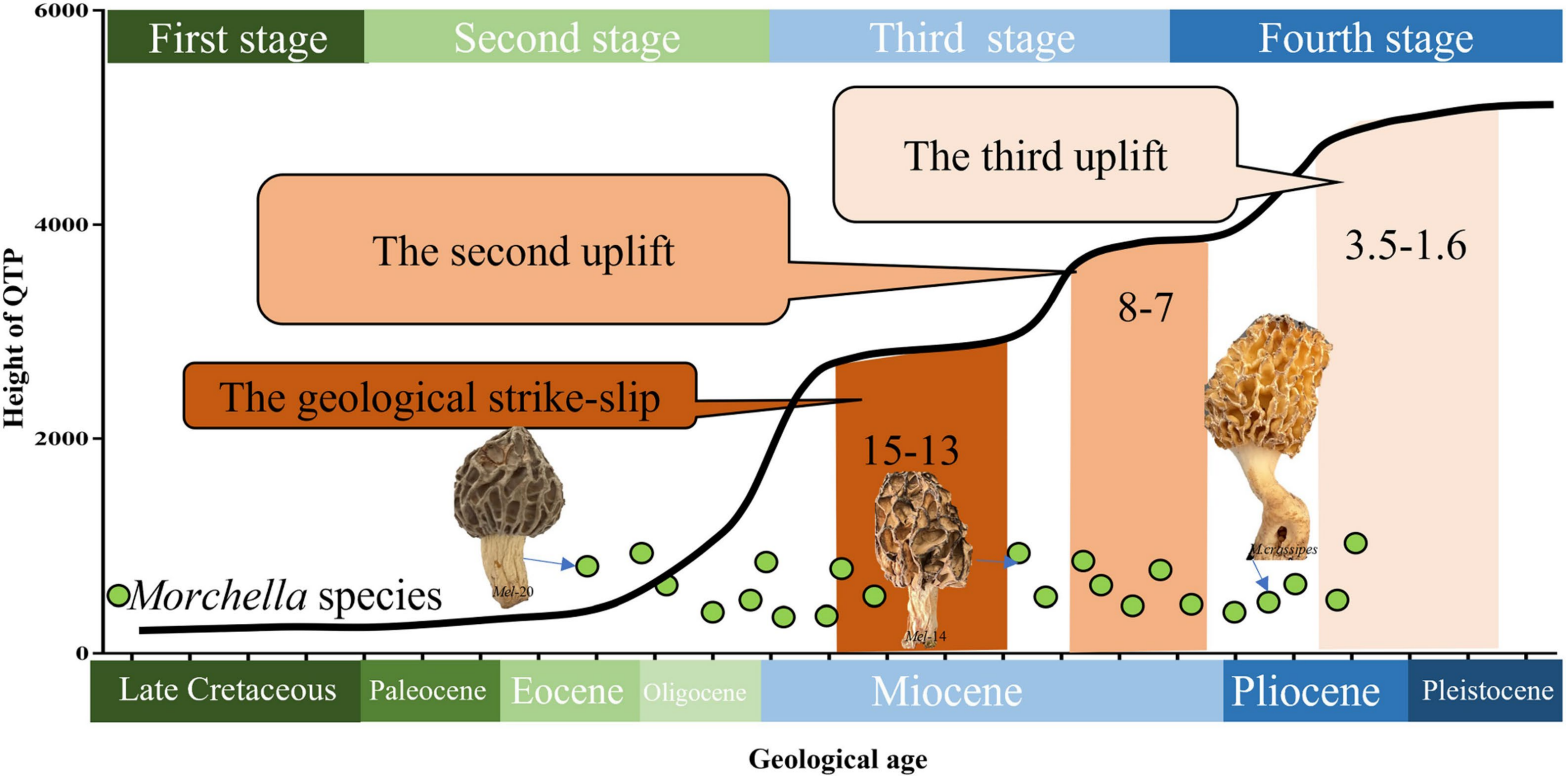
Species differentiation of *Morchella* related to the stage of plate movement and uplift on the QTPs from the Late Cretaceous to the present. The divergence time of *Morchella* phylospecies in QTPs has remained remarkably diverse during its long evolutionary history, ranging from 50.24 to 4.20 Mya (Eocene-Pliocene), when the historical geological uplift and geological tectonic movement were experienced in the QTPs. The plate activity and plateau uplift of QTP resulting from the collision are divided into four stages: (1) Late Cretaceous in the first stage marked as dark green; (2) Tertiary Period in the second stage marked as light green; (3) Neogene in the third stage marked as light blue; (4) Quaternary Period in the fourth stage marked as dark blue. The green dots represent the *Morchella* individual.

The divergence time of 18 *Morchella* phylospecies in the QTPs ranged from 50.24 to 4.20 Mya (Figure 4; Table 1). The earliest diverging branch of *Morchella* in the QTPs was represented by the monotypic *M. rufobrunnea* (originated in North America) lineage with an estimated divergence time of 154.15 Mya (95% HPD interval: 152.14–156.08); the second diverging branch of Esculenta and Elata clades at 98.63 Mya (95% HPD interval: 97.30–100.0); the third evolutionary diversification of the Elata Clade was dated at 57.94–68.97 Mya (95% HPD interval: 30.09–69.04; 40.17–98.45) and the Esculenta Clade at 20.41–20.83 Mya (95% HPD interval: 6.18–45.22; 2.62–52.67). The phylospecies of *M. norvegiensis* = *M. eohespera/Mel*-19, *M. deliciosa/Mel*-13, *Mel-*14, *Morchella eximia*, *M. costata*, *M. esculenta/Mes*-8, *M. crassipes*, and *Mes-*19, were estimated at 50.24 Mya (95% HPD interval: 40.17–98.45), 40.63 Mya (95% HPD interval: 18.95–63.83), 36.41 Mya (95% HPD interval: 2.83–66.27), 25.87 Mya (95% HPD interval: 4.77–48.44), 14.24 Mya (95% HPD interval: 6.18–45.22), 4.20 (95% HPD interval: 0.02–19.54), respectively. In summary, *Morchella* phylospecies in QTPs has maintained a very diversified evolutionary history during the Eocene and Pliocene, when the historical geological uplift and geological tectonic movement were experienced in the QTPs (Figure 5).

**Table 1:** The estimation of the divergence time of *Morchella* in the QTPs.

Node	Individual numbers	Mean ± standard error^a^	95% HPD^a^	Ancestors region^b^	Geological events^c^
Ascomycota/Basidiomycota	–	564.85 ± 0.11	467.24–666.82	–	Cambrian
*Verpa bohemic*/*Morchella*	–	274.03 ± 0.31	272.08–276	–	Triassic
*Morchella rufobrunnea*	–	154.15 ± 0.06	152.14–156.08	North America	Cretaceous
Esculenta/Elata	–	62.95 ± 0.38	51.24–69.76	–	Paleocene
*Morchella eximia*	18	21.6 ± 0.35	6.15–60.04	Qilian Mountains in the eastern part of the QTPs	the geological strike-slip
*Morchella eohespera*	19	50.24 ± 0.25	40.17–98.45		The first stage of the uplift
*Mel*-33	3	16.19 ± 1.07	0.11–52.08		The second uplift
*Morchella sextelata*	8	11.15 ± 1.94	0.1–35.13		The third uplift
*Morchella costata*	11	20.05 ± 0.60	6.82–58.62		the geological strike-slip
*Mel*-14	11	26.41 ± 4.16	2.62–52.67		the geological strike-slip
*Mel*-13	21	40.63 ± 0.97	18.95–63.63		The first stage of the uplift
M*orchella septimelata*	3	26.08 ± 0.71	0.04–54.98		the geological strike-slip
*M. purpurascens*/*Mel*-20	5	45.89 ± 0.55	16.89–88.59		The first stage of the uplift
*M. pulchella*/*Mel-*31	3	33.62 ± 0.50	7.11–74.38		The second stage of uplift
*Morchella esculenta*	24	25.87 ± 0.87	4.77–48.44	Shangri-la in the southwestern QTPs	the geological strike-slip
*Morchella crassipes*	26	5.72 ± 0.15	0.16–16.35		The third uplift
*Morchella spongiola*	11	20.41 ± 2.08	2.62–52.67		the geological strike-slip
*Mes-*26	9	7.8 ± 0.33	0.54–32.68		The third uplift
*Mes-*19	25	24.14 ± 0.31	0.09–28.99		the geological strike-slip
*Mes-*12	4	16.54 ± 0.15	0.02–19.54		The second uplift
*Mes-*9	4	24.83 ± 1.10	0.0–23.54		the geological strike-slip
*Mes-*6	11	7.8 ± 0.33	0.54–32.68		
					The third uplift

a The divergence times and 95% higher posterior densities (HPDs) were generated by molecular clock analysis using the two concatenated datasets (ITS + LSU and *EF1-α + RPB1 + RPB2*) dataset.

b The ancestor region probability was obtained from the most likely states (MLS) using the Bayesian binary method (BBM) and statistical dispersal-vicariance analysis (S-DIVA) as implemented in Reconstruct Ancestral State in Phylogenies (RASP v3.1).

c The geological events were referenced in Dai et al. (2019).

### The reconstruction of the ancestral area and spatial patterns of *Morchella* in the QTPs

**Figure 6:**
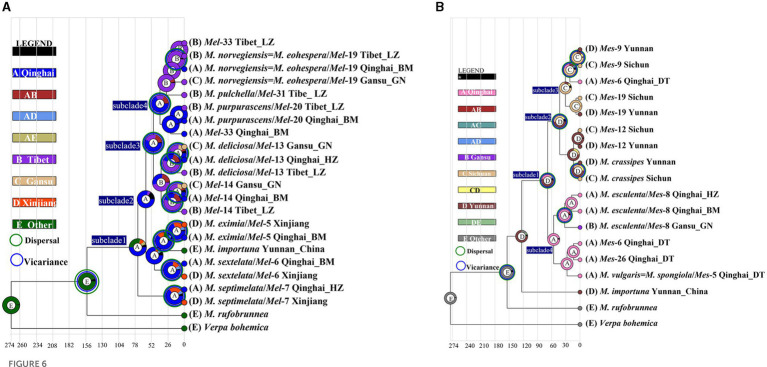
Ancestral area reconstruction of *Morchella* phylospecies in QTPs using the ITS dataset. The chronogram was obtained by molecular clock analysis using BEAST. The pie chart in each node indicates the possible ancestral distributions inferred from Bayesian Binary MCMC analysis (BBM) implemented in RASP. Bayesian credibility values (PP) over 0.85 are indicated near the pie chart of the tree. The green circle around the pie charts indicates possible dispersal events, the blue circle indicates possible vicariance events as suggested by BBM analysis. **(A)** Elata clade; **(B)** Esculenta clade.

**Figure 7:**
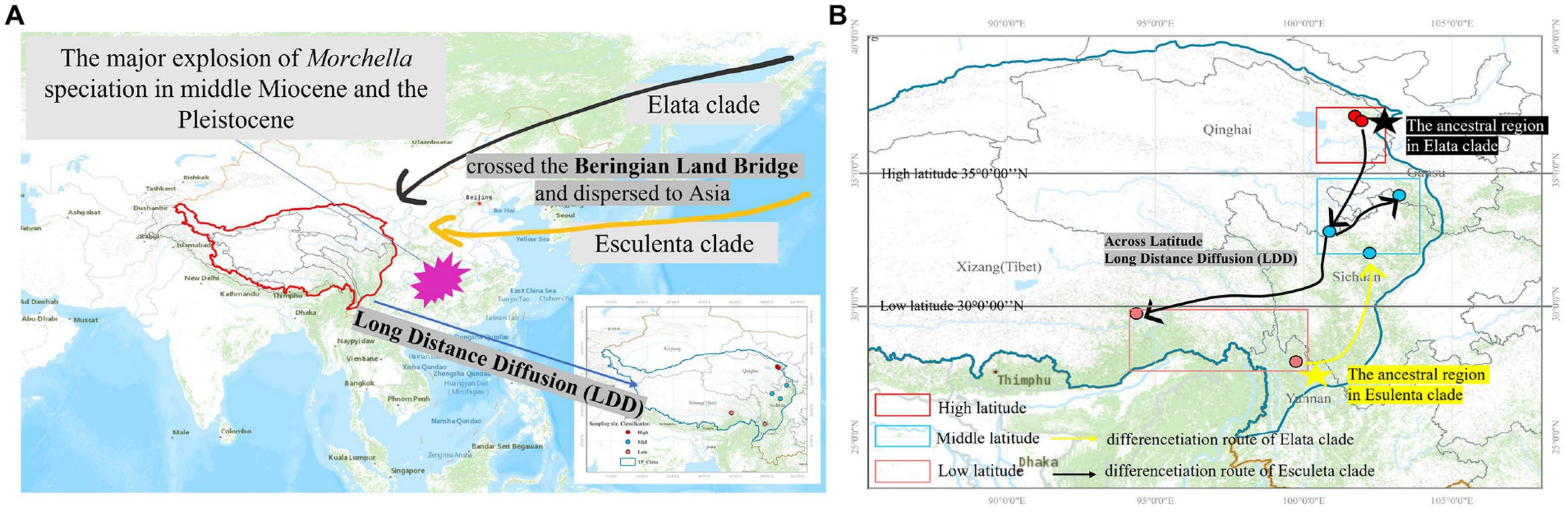
Map of the geographic distribution of *Morchella* and possible dispersal routes in QTPs generated by ArcGIS v10.1. **(A)** The major explosion of *Morchella* speciation in the middle Miocene and the Pleistocene crossed the Beringian Land Bridge and dispersed to Asia. **(B)** A hypothetical schematic depiction of the original locations, the migration routes the speciation of *Morchella* in QTPs.

There were 19 dispersal events and 19 vicariance events that could explain the current distribution of the *Morchella* phylospecies in the QTPs. For the Elata clade, region A (Qilian Shan, Qinghai) located in the eastern part of QTPs, has the highest probability (50.08%) of being the ancestral area during the Eocene (Figure 6A). For the Esculenta clade, region D (Shangri-La, Yunnan), which was located in the southwestern part of QTPs, has the highest probability (77.48%) of being the ancestral area during the Miocene (Figure 6B). In general, based on the phylogeographic structures of the 8 subclades, 3 distribution patterns can be summarized: (i) the wide distribution around the QTPs, such as the *M. deliciosa/Mel*-13, *Mel*-14, *M. norvegiensis* = *M. eohespera/Mel*-19, suggests that not all of the *Morchella* species were narrowly distributed; (ii) the long-distance dispersal with latitude-based structure. (iii) Multi-origin: the two clades have different origins, the Easculenta clade originated from Shangri-La while the Esculenta clade originated from Qilian Shan (Figure 7).

### The origination and evolution of worldwide *Morchella*

**Figure 8:**
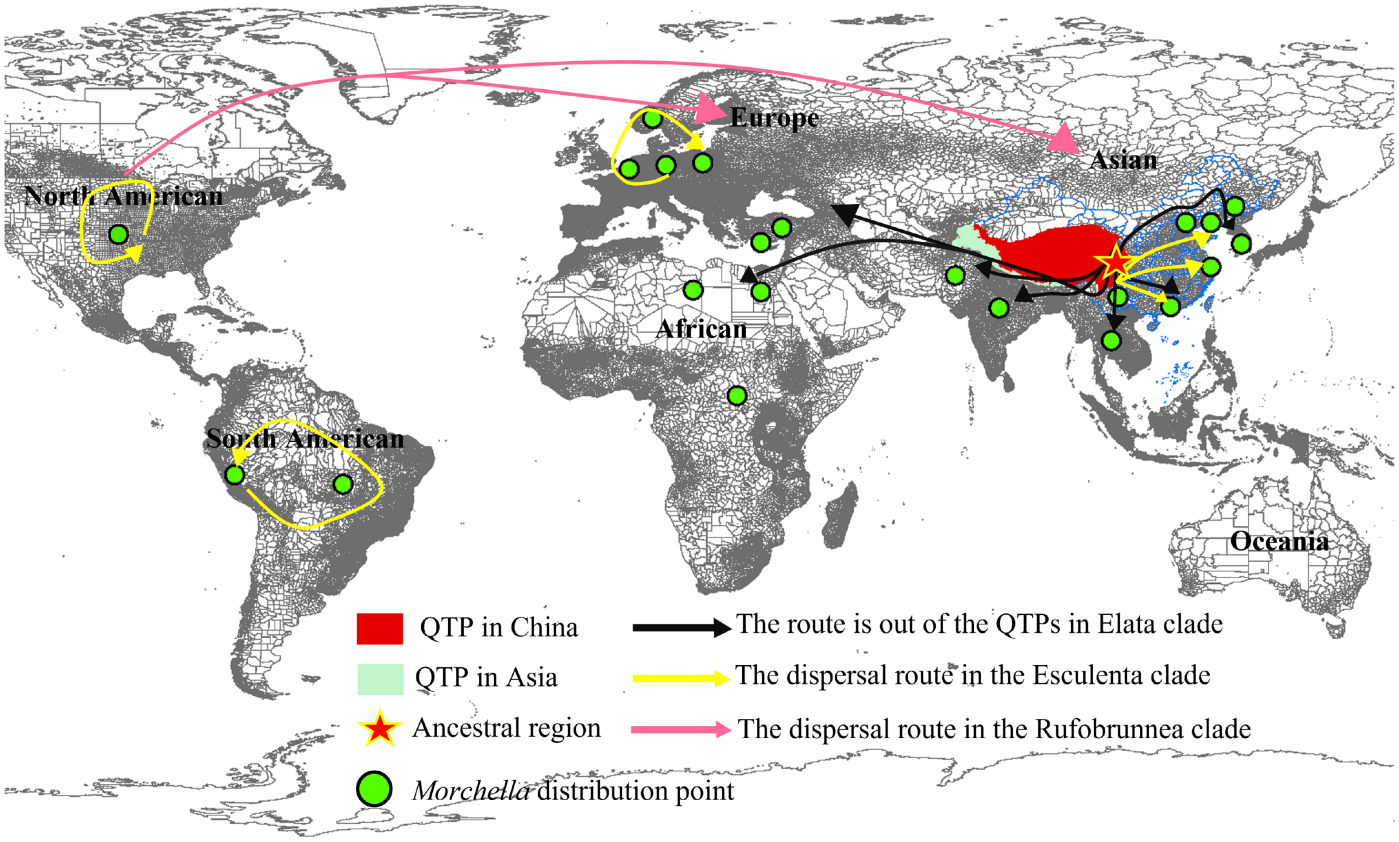
The origin of the QTPs and the route out of the QTPs in *Morchella. Morchella* originated from Qilian Shan and spread along the Himalayas on the edge of QTPs to surrounding Europe and South Africa in the Elata clade (Black route). Each continent has its ancestor in the Esculenta clade (Yellow route).

For the *Morchella* genus, there is no Rufobrunnea clade on any continent except North America, and most of the species that are widely distributed in the continental region (besides Oceania) have the earliest divergence time in the QTPs, the ancestor of *Morchella* both the Esculenta and Elata probably originated in QTPs and initially covered Eurasian and South Africa in the Early Tertiary (Figure 8; Table 2).

**Table 2:** Species initiation differentiation time and ancestor region reconstruction of worldwide *Morchella* species.

No	Phylospecies	Distribution^a^	Extinction event time (95% HPD interval)^b^	Initiation divergence time (95% HPD interval)^c^	Probability of ancestor region^d^		
					QTPs (%)	Europe (%)	North America (%)
1	*M. spongiloa*	Asia, Europe, and North America	68.70 Mya (66.70–70.63)	49.03 Mya (26.48–68.63)	16.23	6.93	none
2	*M. esculenta*	Asia and North America	59.98 Mya (26.48–68.63)	38.92 Mya (19.26–56.02)	38.81	20.10	5.91
3	*M. crassipes*	Asia, Europe, North America, South America, South Africa	65.72 Mya (59.87–70.12)	68.97 Mya (44.12–66.38)	23.45	25.86	39.67
4	*M. eohespera*	Asia, Central Europe, North America	28.00 Mya (4.11–59.68)	52.25 Mya (24.37–74.35)	76.92	6.50	7.50
5	*Mel*-13	Asia, Europe	35.67 Mya (10.54–62.42)	19.59 Mya (0.50–44.58)	0	10.67	none
6	*Mel*-14	Asia, Europe	39.55 Mya (12.55–42.72)	39.55 Mya (12.55–42.72)	33.40	8.30	none
7	*M. eximia*	Asia, Europe, and North America	61.89 Mya (43.31–74.34)	37.78 Mya (10.24–67.87)	32.64	39.96	17.22
8	*M. costata*	Asia, Europe	72.64 Mya (71.81–75.89)	72.64 Mya (71.81–75.89)	81.35	1.87	none

a Searched in NCBI.

b The divergence times were generated by molecular clock analysis using the ITS dataset.

c The means and 95% higher posterior densities (HPDs) of age estimates were obtained from the combined outputs using Tracer.

d The ancestor region probability was obtained from the most likely states (MLS) using RASP.

For the Esculenta clade: (1) *M. vulgaris* = *M. spongiola/Mes*-5 there were distributed in central Europe and Asia, and the initial differentiation of *M. spongiloa* in these two areas was 28.88 Mya (95% HPD interval: 4.11–59.68) and 49.03 Mya (95% HPD interval: 26.48–68.63), respectively. They went through an extinction event between Asia and Europe. QTPs of Asia and Germany of Europe were most likely ancestral regions. However, the Asian phylospecies diverged 11 Mya earlier than the European species, and the QTPs were the ancestral region of *M. spongiloa* (Table 2; Supplementary Figure S1). (2) *M. esculenta/Mes*-8 was widely distributed in Europe, North America, and Asia, among them, the earliest divergences of this species occurred in China at 38.92 Mya (95% HPD interval: 19.26–56.02). That phylospecies experienced one extinction event in the distribution area and the highest probability (38.81%) of being a putative ancestral region is the QTPs (Table 2; Supplementary Figure S2). (3) *M. crassipes* is a widely distributed species in Asia, Europe, North America, and South Africa. The earliest differentiation of this species is Shanghai during the 68.97 Mya (95% HPD interval: 67.04–70.93), but North America has the highest probability (39.67%) of being a putative ancestral region (Table 2; Supplementary Figure S3).

For Elata clade: (4) *M. norvegiensis* = *M. eohespera/Mel*-19 (Elata clade), were distributed in Central Europe, Asia North America. The initial divergence time in the QTPs is 52.25 Mya (95% HPD interval: 24.37–74.33), which is earlier than in North America and Central Europe [15.81Mya (95% HPD interval: 0.13–49.54), 9.79Mya (95% HPD interval: 0–36.55)]. The QTPs have the highest probability (76.92%) of being the ancestral area of *M. eohespera* and the dispersal events and extinction events also occurred in the continental range (Table 2; Supplementary Figure S4). (5) *M. deliciosa/Mel*-13 and *Mel*-14 are widely distributed in Eurasia, especially in the QTPs. *M. deliciosa/Mel*-13 and *M. importuna* diverged at 35.67 Mya (95% HPD interval: 21.92–72.25), while *Mel*-14 and *M. importuna* diverged at 39.55Mya (95% HPD interval: 12.05–72.82). Europe is the putative ancestral region of *Mel*-13 (10.67%) while QTPs have the highest probability of being an ancestral region of *Mel*-14 (33.40%), and they all experienced one extinction event (Table 2; Supplementary Figure S5). (6) *M. eximia* are widely distributed in America, Europe, and Asia, which were estimated at 16.56 Mya (95% HPD interval: 10.23–67.80) in QTPs (Supplementary Figure S6). (7) *M. costata* were widely distributed in Eurasia. The initial differentiation in QTPs was 40.53 Mya (95% HPD interval: 42.31–71.50), which has the highest probability (81.35%) of being an ancestral region of *M. costata* (Table 2; Supplementary Figure S7). Overall, six of the eight worldwide *Morchella* originated in the QTPs, the ancestral region of 60% (3/5) of the Elata clade and 66.66% (2/3) of the Esculenta clade were the QTPs; therefore, it is possible that QTPs was the center of origin for the current *Morchella* species diversity. *Morchella* originated from the QTPs and expanded out and spread to the other continents in Paleogene Period (Figure 8).

## Discussion

This is the first time report the phylospecies diversity of *Morchella* in the QTPs used GCSPR, and the results indicate that there is 18 phylospecies of *Morchella* in QTPs, which far exceeds the number of published taxa for these areas (Du et al., 2012b, 2016, 2019). Generally, both Esculenta and Elata clades have preferred habitats, known as phylogenetic niche conservation (PNC; Donoghue, 2008). We investigated the occurrence and preferred habitats of *Morchella* in the QTPs and found that occurred in primary forests with high vegetation coverage, deciduous forests, or mixed coniferous and broad-leaved forests, such as the primary forests of Qilian Shan, Belong River, Mote, and Shangri-la, all of which belong to the eastern or northwestern margin of the QTPs. In addition, about 90% of the species are found in temperate deciduous forests, while 80% of the species were found in coniferous forests (Figure 1). It can be inferred that the occurrence of *Morchella* in the QTPs was accompanied by the establishment of the temperate deciduous biome and coniferous biome in the late Cretaceous (O’Donnell et al., 2011; Du et al., 2012a, 2015; Chen et al., 2015).

The maximum crown age of *Morchella* in the QTPs was estimated to be around the Late Cretaceous (98.62 Mya). The majority of intracontinental range expansions within *Morchella* in the QTPs appear to have taken place relatively recently between the middle Oligocene and Miocene. During that time, the uplift of the southwest block (Yunnan-Guizhou Plateau) and the eastern margin of the QTP resulted in the distribution of plant diversity in this region. Previous studies have shown that the Beringian Land Bridge (BLB) plays a key role in the spread of *Morchella* species from North America to Asia in the early Cretaceous, which was a natural route linking Eurasia and North America (O’Donnell et al., 2011; Du et al., 2012a, 2015; Loizides et al., 2016, 2021). Palaeobotanical data indicate that the BLB route played a crucial role in plant dispersal for *Populus*, *Lonicera*, *Leibnitzia*, and *Linnaea* (Wang et al., 2009; Qiu et al., 2011; Wen et al., 2014; Du et al., 2015; Wang et al., 2015; Wu et al., 2017). The divergence events exhibited by the plant taxa (*Pinus*, *Abies*, and *Picea*) confirm the geological events associated with species diversification, and those indigenous florae provide native secluded habitats for ancestral species of *Morchella* in QTPs (O’Donnell et al., 2011; Loizides et al., 2021). The Beringian Land Bridge as a species expansion channel is a critical driver of *Morchella* migrating to the QTPs between the Cretaceous and Paleocene when plant diversity was already established on QTPs, which means that the QTPs had formed a variety of suitable habitats for living organisms before in the Paleocene.

The differentiation of the *Morchella* is strongly linked to the geological movements of the QTPs (Figure 5). Palaeogeographical evidence suggests that plate activity and plateau uplift of QTP resulting from the collision are divided into four stages (Shi et al., 1998; Ding et al., 2022). (i) Late Cretaceous, the ancient Mediterranean crust subducted to the Eurasian continental crust, blowing the prelude to the plateau uplift. At this stage, *Morchella* of western North America is diverging from its closest relatives in the early Cretaceous, which are divided into Rufobrunnea, Esculenta, and Elata clades, and palaeogeological events played a driving role in the dispersal of *Morchella* (O’Donnell et al., 2011). (ii) Tertiary Period (Including the Paleocene, Eocene, and Oligocene), the Indian plate collided with the Eurasian plate, opening the prelude to the plateau uplift. The Indian plate rapidly drifted northward, the Indo-Pak subcontinent and the subcontinent were getting closer, and the ancient Mediterranean crust gradually disappeared during the Oligocene. In our data, *M. norvegiensis* = *M. eohespera/Mel*-19 was differentiated at 50.24 Mya with the new uplift belts of Tengchong-Bango formatted and the uplift area of Songpan-Ganzi shrank to the east during Eocene (Wang et al., 2008, 2022). Eocene (55.8 ± 0.2) was a key period for Asian paleoenvironmental changes and was characterized by a warmer climate than any other interval in the Cenozoic (Shi et al., 1998; Zachos et al., 2001; Hoorn et al., 2012). *M. norvegiensis* = *M. eohespera/Mel*-19 in the middle latitudes region were differentiated at 34.24 Mya with the further uplifted of Kunlun-Algin-Qilian during the Oligocene, which has been called the beginning of the present ‘icehouse’ epoch (Ling et al., 2021; Dieter et al., 2022). (iii) Neogene, the Indian and Tarim plates compressed and subducted to the QTPs with greater stress, and the plateau was greatly uplifted, forming the QTPs and the Himalayas. Our results indicate that 9 of the 18 QTPs *Morchella* species lineages (i.e., 50%) diversified between the middle Miocene and the present (Figure 4). (iv) Quaternary Period, the plateau was greatly uplifted, forming the present towering QTP. The Esculenta clade of *Morchella* in the QTPs was undergoing differentiation at this time. Tectonic activity and climate change in geological periods can form geographical isolation barriers to promote species differentiation and increase diversity, and can also reduce biological diffusion barriers to expand species distribution areas and increase biological exchanges between different regions (Che et al., 2010; Antonelli et al., 2018; Rahbek et al., 2019; Ding et al., 2020). Generally speaking, the formation and evolution of *Morchella* phylospecies in the QTPs were affected by the tectonic uplift of the QTPs and the geological movements of QTPs were an important force for the differentiation of the *Morchella* species (Merckx et al., 2015; Dai et al., 2019). The early marginal geological movement of the QTPs caused strong habitat fragmentation and rapid expansion of dry and cold habitats, which further strengthened the monsoon climate in East Asia and promoted the differentiation of microorganisms in the QTPs (An et al., 2001; Yang, 2005; Wan et al., 2014; Liu et al., 2016).

At least three geographic distribution patterns were discovered to correspond to the following phylogeographic structures. (i) the wide distributions around the QTPs: geographic locations of 4 endemic species, such as *Mel*-13, *Mel*-14, *Mel*-19, and *M. esculenta*, cross over three regions (Qinghai, Gansu, and Tibet in high, middle, and low latitudes respectively) of the QTPs. (ii) provincialism in the QTPs: the specific local distributions of two species in the Elata clade (*M. pulchella/Mel*-31, *M. purpurascens/Mel*-20) were unique (only in the Tibet region). (iii) the long-distance dispersal with latitude-based distributions: the divergence of *Mel*-13 and *Mel*-14 experienced two dispersal and vicariance events in three regions of the QTPs (Figure 6A). *Mel*-13 diverged in the mid-latitudes at 40.63 Mya and subsequently differentiated to the higher-latitudes at 34.76 Mya, which was based on latitude long-distance dispersal. *Mes*-19 also experienced dispersal and vicariance events in mid-latitudes and lower-latitudes, although this species originated from low latitudes (Yunnan) of the southwestern QTP (Figure 6B). To sum up, long-distance dispersals based on latitudes may have contributed to the current disjunct distribution ranges of *Morchella*, which is supported by biogeographic studies of plants and animals on the QTPs (Figure 7). The spread and differentiated way of *Morchella* in the global range, including intercontinental, intracontinental, and putative transoceanic long-distance dispersals, was slightly different from our result in QTPs (Du et al., 2012; Quan et al., 2014).

The complex topography and a special eco-climate of QTPs provide multiple periglacial microrefugia for *Morchella*. The Qilian Shan, located in the northeastern of the QTP, was defined as the ancestral region of the Elata clade as early as the Eocene. The Qilian Shan is an intraplate orogenic belt that experienced multiple episodes of fold and thrust deformation throughout the Mesozoic and Cenozoic periods, which controlled the evolution of regional climatic conditions in a broad region in inland Asia since the Miocene (Jia et al., 2022). The Shangri-La region in the southeast of the QTPs, which was the ancestors’ region for the Esculenta clade of *Morchella*, not only served as an important glacial refugium but also as a center of diversification for a variety of plants and animals (Suzuki et al., 2003; Yang et al., 2008, 2009; Harrison and Noss, 2017). The QTPs contain several important biodiversity hotspots, particularly along its southeastern margin (the Henduan Mountains and the eastern Himalayas), which were proposed to be glacial refugia for alpine hepialid species that effectively avoided extinction during the Quaternary glacial period (Hewitt, 2000; Dai et al., 2019).

Multiple pieces of evidence suggest that the Elata clade originated from QTPs, whereas the origin of the Esculenta clade was diverse. Our results show that the worldwide distribution species was an initial differentiation time of 73.78 Mya–35.67 Mya earlier than Central Asia, Europe, and South Africa (Figure 8). During the Early Tertiary (65.5–23.03 Mya), the Indian plate collided with the Eurasian plate opening the prelude to the plateau uplift, which is the emergence time of modern organisms (Liu and Hu, 2003). For example, in *M. norvegiensis* = *M. eohespera/Mel*-19, the divergence time was estimated at 52.25 Mya, which was earlier than that of Europe (Supplementary Figure S4). The *Morchella* was out of QTPs probably because of Eurasia’s collision and plate migration. On the other hand, the probability of QTPs as the most likely ancestral region for these widely distributed species is greater than that of Europe, North America, and South Africa (Table 2). All world-widely phylospecies have experienced historical extinction events that may be caused by the palaeogeology events and tectonic movement of the QTPs during the Eocene, followed by the differentiation and expansion of the two clades of *Morchella* (Figure 8). For the Elata clade, *Morchella* originated from Qilian Shan and spread along the Himalayas on the edge of QTPs to surrounding Europe and South Africa. However, after the Esculenta clade originated from Shangri-La and spread to other regions of Asia, it was subsequently blocked by the geographical isolation formed in the Quaternary, which led to the diversification of the origin of the Esculenta clade. This result further enriches the theory of species origin in QTPs, except for the reported species such as *Floccularia luteovirens*, *Ophiocordyceps sinensis*, *Saccharomyces pastorianus*, etc. (Quan et al., 2014; Dai et al., 2019; Wu Y. et al., 2021; Bai, 2022; Guo et al., 2022).

## Conclusion

In this study, a total of 216 individuals were identified 18 *Morchella* phylospecies of two clades (Elata and Escuelnta clade), and the results indicate that the *Morchella*’s phylogenetic diversity within the QTPs far exceeds the number of published taxa for these areas. The divergence time of the 18 *Morchella* phylospecies occurred in the Eocene-Pliocene period (50.24–4.2 Mya), it was strongly related to the geological strike-slip and the uplift movements of the QTPs, suggesting that the geographical movements had a large influence on the differentiation of the *Morchella*. Furthermore, the reconstructed ancestral areas of the Elata and Esculenta clades indicate that the northwestern and southeastern regions of QTPs are the likely ancestral area, which also has been suggested to coincide with the glacial refugia in the Quaternary. Moreover, we confirmed that Elata and Esculenta of *Morchella* originated from QTPs and spread out with plateau geological uplift, transoceanic or transcontinental long-distance dispersal, orogeny, or geographical movements during the Cenozoic Era. The origin of the Esculenta clade is not unique. These results offer strong evidence for the theory of the origin of species in the QTPs.

## Data availability statement

The data presented in the study are deposited in the NCBI repository, accession number of those data was list in Supplementary Table S1.

## Author contributions

QM: conceptualization, methodology, and writing-original draft. ZX: supervision, validation, and writing review and editing. HX, JG, and QP and TM: writing-review and editing. YT, BW, YM, SY, JY, YD, FZ, and TG: investigation. All authors contributed to the article and approved the submitted version.

## Funding

This work was supported by the Natural Science Planning Project of Qinghai Province (Grant no. 2021-HZ-802), and the first batch of central forestry and grassland ecological protection and restoration funds in 2021 (2021-87).

## Conflict of interest

The authors declare that the research was conducted in the absence of any commercial or financial relationships that could be construed as a potential conflict of interest.

## Publisher’s note

All claims expressed in this article are solely those of the authors and do not necessarily represent those of their affiliated organizations, or those of the publisher, the editors and the reviewers. Any product that may be evaluated in this article, or claim that may be made by its manufacturer, is not guaranteed or endorsed by the publisher.

## Footnotes

^1^http://iqtree.cibiv.univie.ac.at/

^2^https://itol.embl.de/personal_page.cgi

^3^https://itol.embl.de/personal_page.cgi
