# Corrigendum: Out of the Qinghai-Tibetan plateau: origin, evolution and historical biogeography of *Morchella* (both Elata and Esculenta clades)

**DOI:** 10.3389/fmicb.2023.1222851

**Published:** 2023-08-07

**Authors:** Qing Meng, Zhanling Xie, Hongyan Xu, Jing Guo, Yongpeng Tang, Ting Ma, Qingqing Peng, Bao Wang, Yujing Mao, Shangjin Yan, Jiabao Yang, Deyu Dong, Yingzhu Duan, Fan Zhang, Taizhen Gao

**Affiliations:** ^1^College of Ecological and Environment Engineering, Qinghai University, Xining, Qinghai, China; ^2^State Key Laboratory Breeding Base for Innovation and Utilization of Plateau Crop Germplasm, Qinghai University, Xining, Qinghai, China; ^3^Academy of Agriculture and Forestry Sciences, Qinghai University, Xining, Qinghai, China; ^4^State-owned Forest Farm of Tianjun County, Delingha, Qinghai, China; ^5^Forestry and Grassland Station of Tianjun County, Delingha, Qinghai, China

**Keywords:** *Morchella*, Qinghai-Tibet plateau subkingdoms, multigene phylogenetics, age estimation, phylogeographic structure

In the published article, there was an error in the legend for Figure 2C and Figure 3C as published. The names of various *Morchella* phylospecies were incorrectly presented. The corrected parts of the legends appear below.

**Figure 2:**
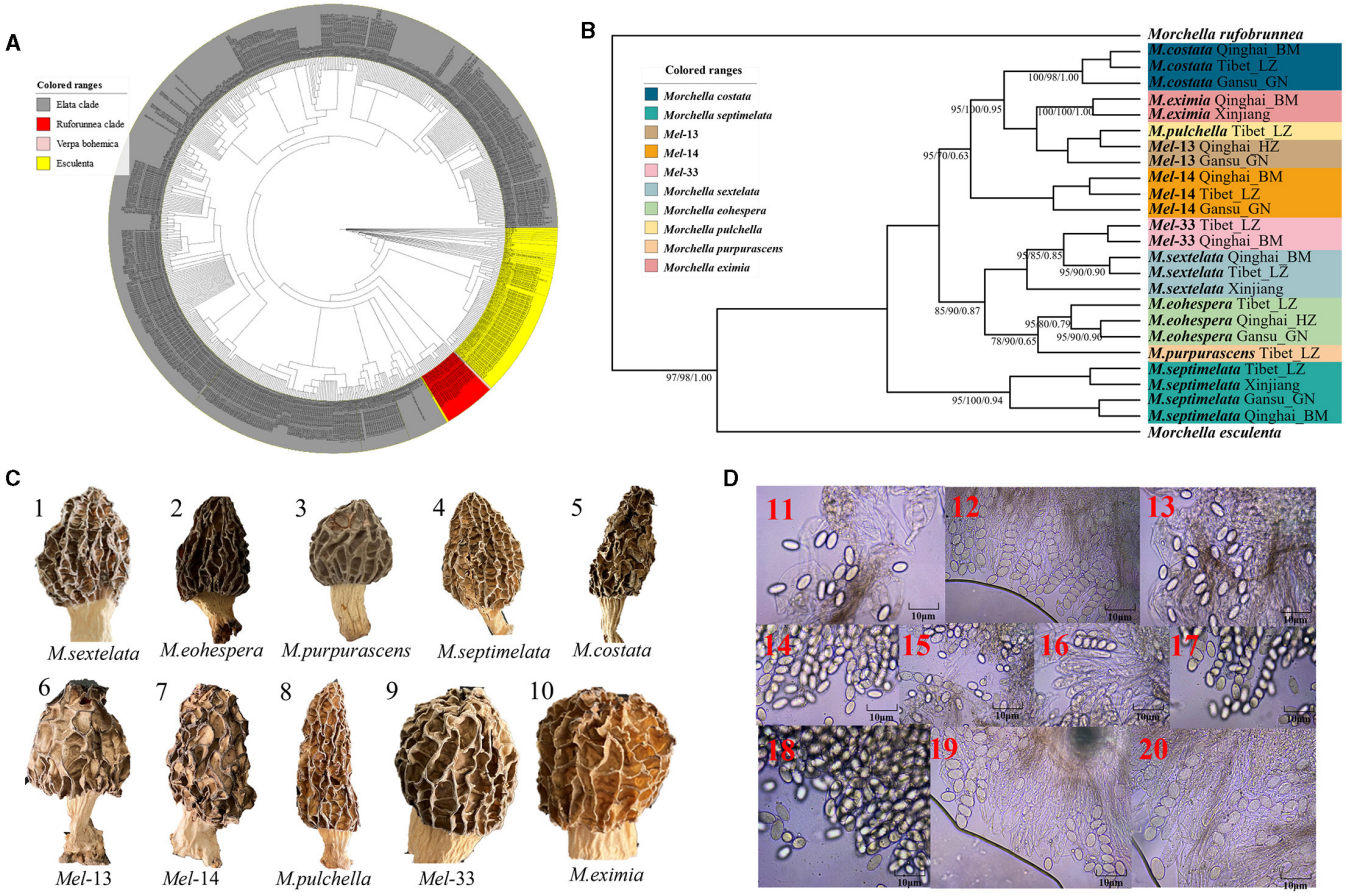
Species recognition of the *Morchella* in Elata clade from the QTPs. **(A)** Bayesian inference phylogenetic analyses of the Elata clade were inferred from 115 internal transcribed spacer (ITS) sequences representing a total of 10 phylospecies. **(B)** Phylogenetic analyses of the Elata clade were inferred from 120 (24^*^5) multi-genes (ITS + LSU + *EF1-*α + *RPB1* + *RPB2*) sequences representing a total of ten phylospecies. Branches are labeled where MP/ML support is greater than 60% and collapsed below that support threshold. BPP is labeled were greater than 0.95. **(C)** Morphological diversity of the 10 Elata clades' ascocarps from the QTPs: *M. sextelata*/*Mel-*6 (1), *M. norvegiensis* = *M. eohespera/Mel-*19 (2), *M. purpurascens*/*Mel*-20 (3), *M. septimelata*/*Mel*-7 (4), *M. costata* (5), *M. deliciosa*/*Mel*-13 (6), *Mel*-14 (7), *M. pulchella*/*Mel*-31 (8), *Mel*-33 (9), *M. eximia*/*Mel*-5 (10). **(D)** Micromorphological ascospores of the 10 Elata clades.

**Figure 3:**
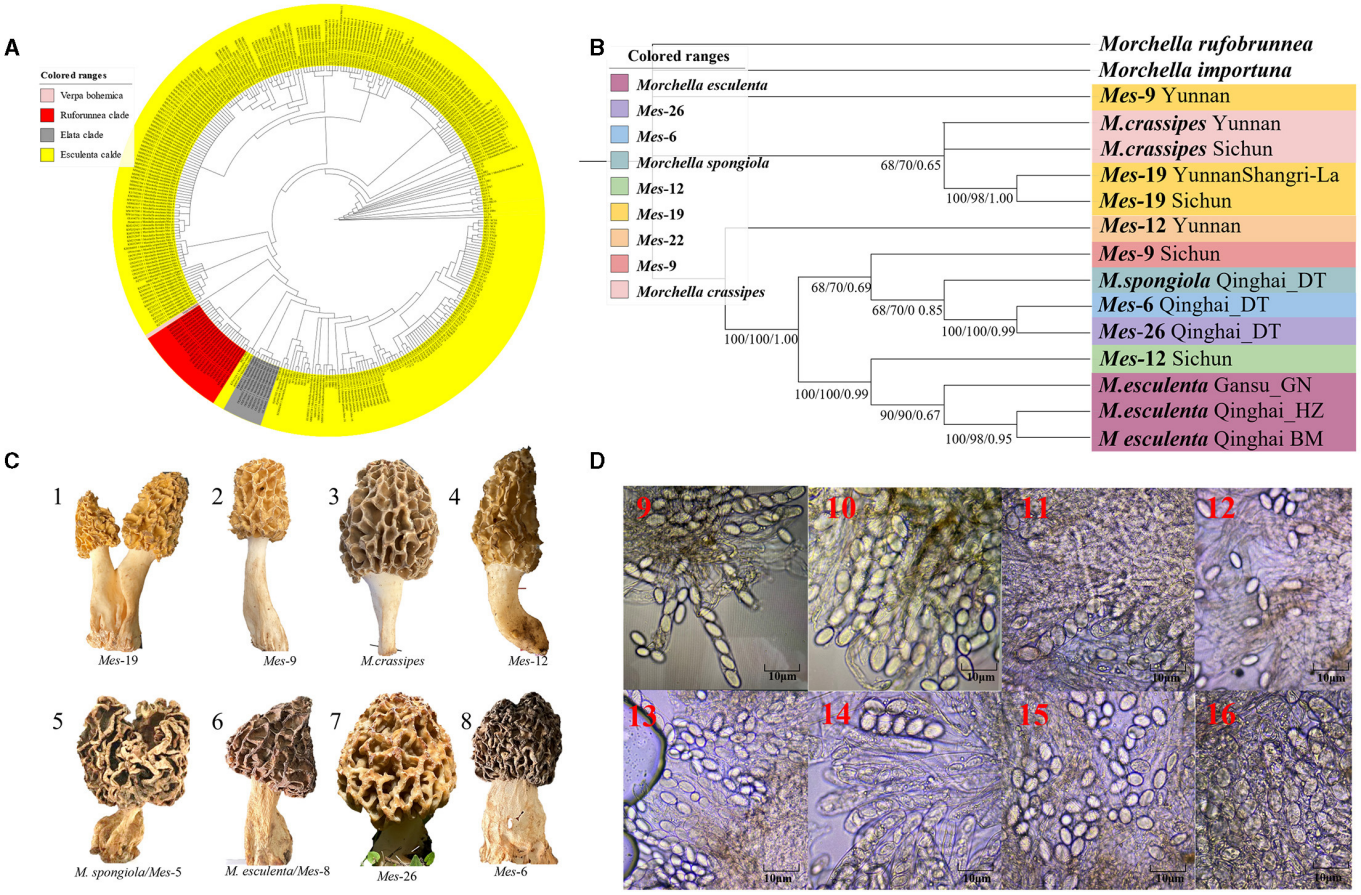
Species recognition of the *Morchella* in Esculenta clade from the QTPs. **(A)** Bayesian inference phylogenetic analyses of the Esculenta clade were inferred from 101 ITS sequences representing a total of 8 phylospecies. **(B)** Phylogenetic analyses of the Esculenta clade were inferred from 70 (14^*^5) multi-genes (ITS + LSU + *EF1-*α + *RPB1* + *RPB2*) sequences representing a total of 8 phylospecies. Branches are labeled where MP/ML support is greater than 60% and collapsed below that support threshold. BPP is labeled were greater than 0.95. **(C)** Morphological diversity of the 8 Esculenta clades' ascocarps from the QTPs: *Mes*-19 (1); *Mes*-9 (2), *M. crassipes* (3), *Mes*-12 (4), *M. vulgaris* = *M. spongiola*/*Mes*-5 (5), *M. esculenta*/*Mes-*8 (6), *Mes*-26 (7), *Mes*-6 (8). **(D)** Micromorphological ascospores of the 8 Esculenta clades.

Figure 2. Species recognition of the *Morchella* in Elata clade from the QTPs. **(C)** Morphological diversity of the 10 Elata clades' ascocarps from the QTPs: *M. sextelata*/*Mel-*6 (1), *M. norvegiensis* = *M. eohespera/Mel-*19 (2), *M. purpurascens*/*Mel*-20 (3), *M. septimelata*/*Mel*-7 (4), *M. costata* (5), *M. deliciosa*/*Mel*-13 (6), *Mel*-14 (7), *M. pulchella*/*Mel*-31 (8), *Mel*-33 (9), *M. eximia*/*Mel*-5 (10).

Figure 3. Species recognition of the *Morchella* in Esculenta clade from the QTPs. **(C)** Morphological diversity of the 8 Esculenta clades' ascocarps from the QTPs: *Mes*-19 (1); *Mes*-9 (2), *M. crassipes* (3), *Mes*-12 (4), *M. vulgaris* = *M. spongiola*/*Mes*-5 (5), *M. esculenta*/*Mes-*8 (6), *Mes*-26 (7), *Mes*-6 (8).

In the published article, in Figures 2–4, 6 and Table 1 the names of several *Morchella* phylospecies were incorrectly presented.

The corrected Figures 2–4, Figure 6 and Table 1 and their captions appear below.

**Figure 4:**
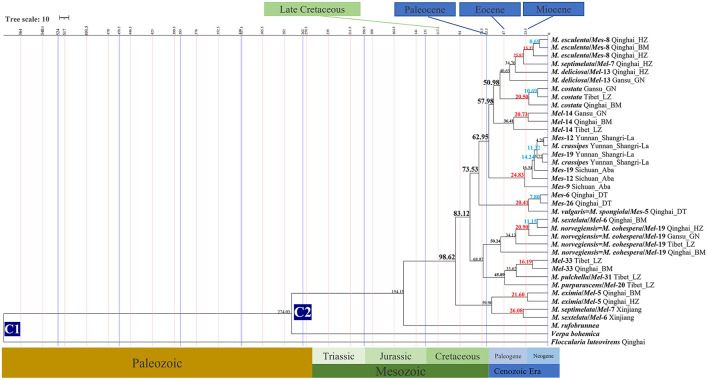
Chronogram and estimated divergence times of *Morchella* in QTPs generated by molecular clock analysis using the two concatenated datasets (ITS + LSU and *EF1-*α + *RPB1* + *RPB2*) dataset. The chronogram was obtained using the Ascomycota-Basidiomycota divergence time of 582.08 Mya as the calibration point 1. The *Morchella*-*Verpa bohenica* divergence time of 274.06 Mya as the calibration point 2. The calibration point and objects of this study are marked in the chronogram. The geological time scale is millions of years ago (Mya). The red font is defined as the first uplift of the QTPs, and the blue font is defined as the second uplift of the QTPs.

**Figure 6:**
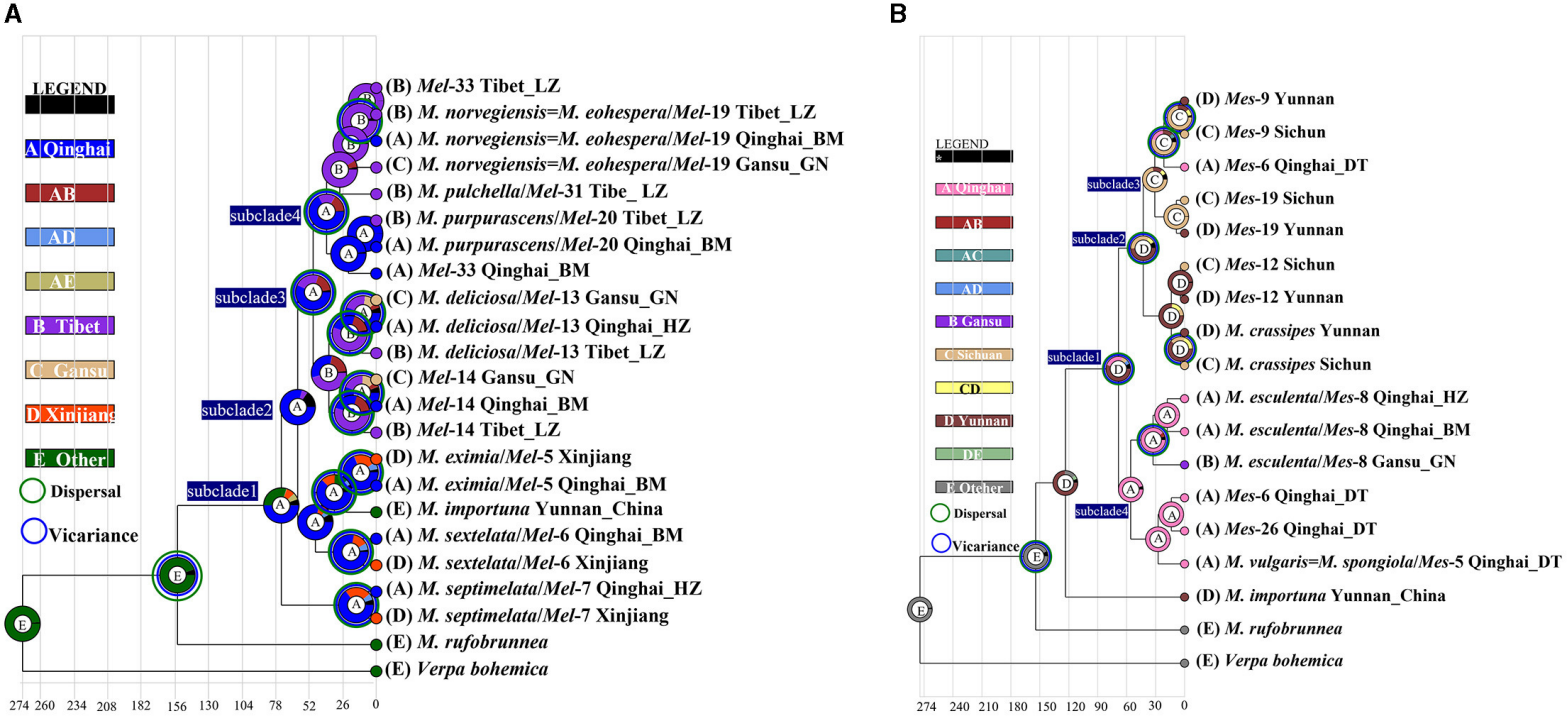
Ancestral area reconstruction of *Morchella* phylospecies in QTPs using the ITS dataset. The chronogram was obtained by molecular clock analysis using BEAST. The pie chart in each node indicates the possible ancestral distributions inferred from Bayesian Binary MCMC analysis (BBM) implemented in RASP. Bayesian credibility values (PP) over 0.85 are indicated near the pie chart of the tree. The green circle around the pie charts indicates possible dispersal events, the blue circle indicates possible vicariance events as suggested by BBM analysis. **(A)** Elata clade; **(B)** Esculenta clade.

**Table 1:** The estimation of the divergence time of *Morchella* in the QTPs.

**Node**	**Individual numbers**	**Mean** ±**standard error**^a^	**95% HPD** ^a^	**Ancestors region** ^b^	**Geological events** ^c^
*Ascomycota/Basidiomycota*	-	564.85 ± 0.11	467.24-666.82	-	Cambrian
*Verpa bohemic/Morchella*	-	274.03 ± 0.31	272.08-276	-	Triassic
*Morchella rufobrunnea*	-	154.15 ± 0.06	152.14-156.08	North America	Cretaceous
*Esculenta/Elata*	-	62.95 ± 0.38	51.24-69.76	-	Paleocene
*Morchella eximia*	18	21.6 ± 0.35	6.15-60.04		The geological strike-slip
*Morchella eohespera*	19	50.24 ± 0.25	40.17-98.45		The first stage of the uplift
*Mel-33*	3	16.19 ± 1.07	0.11-52.08		The second uplift
*Morchella sextelata*	8	11.15 ± 1.94	0.1-35.13		The third uplift
*Morchella costata*	11	20.05 ± 0.60	6.82-58.62	Qilian Mountains in the eastern part of the QTPs	The geological strike-slip
*Mel-14*	11	26.41 ± 4.16	2.62-52.67		The geological strike-slip
*Mel-13*	21	40.63 ± 0.97	18.95-63.63		The first stage of the uplift
*Morchella septimelata*	3	26.08 ± 0.71	0.04-54.98		The geological strike-slip
*M. purpurascens/Mel-20*	5	45.89 ± 0.55	16.89-88.59		The first stage of the uplift
*M. pulchella*/*Mel-*31	3	33.62 ± 0.50	7.11-74.38		The second stage of uplift
*Morchella esculenta*	24	25.87 ± 0.87	4.77-48.44		The geological strike-slip
*Morchella crassipes*	26	5.72 ± 0.15	0.16-16.35		The third uplift
*Morchella spongiola*	11	20.41 ± 2.08	2.62-52.67		The geological strike-slip
*Mes-26*	9	7.8 ± 0.33	0.54-32.68	Shangri-la in the southwestern QTPs	The third uplift
*Mes-19*	25	24.14 ± 0.31	0.09-28.99		The geological strike-slip
*Mes-12*	4	16.54 ± 0.15	0.02-19.54		The second uplift
*Mes-9*	4	24.83 ± 1.10	0.0-23.54		The geological strike-slip
*Mes-6*	11	7.8 ± 0.33	0.54-32.68		The third uplift

a The divergence times and 95% higher posterior densities (HPDs) were generated by molecular clock analysis using the two concatenated datasets (ITS + LSU and *EF1*-α+ *RPB1* + *RPB2*) dataset.

b The ancestor region probability was obtained from the most likely states (MLS) using the Bayesian binary method (BBM) and statistical dispersal-vicariance analysis (S-DIVA) as implemented in Reconstruct Ancestral State in Phylogenies (RASP v3.1).

c The geological events were referenced in Dai et al. (2019).

In Supplementary Figures S1, S4, S5, and Table S1, the names of several *Morchella* phylospeices were incorrectly presented. The corrected Supplementary material accompanies this article.

A correction has been made to the **Results** section, paragraph 1. This sentence previously stated:

“A total of 101 individuals clustered with 10 phylogenetic species, including *Mel*-14, *Mel*-13, *Morchella eohespera*/*Mel*-19, *Morchella eximia*/*Mel*-5, *Morchella costata, Morchella sextelata*/*Mel*-6, *Morchella septimelata*/*Mel*-7, *Morchella purpurascens*/*Mel*-20, *Mel*-33, and *Morchella pulchella*/*Mel*-12 belongs to Elata clade (Figures 2A–C); and a total of 101 individuals clustered with 8 phylogenetic species, including *Morchella spongiola, Mes*-9, *Mes*-12, *Mes*-26, *Morchella crassipes, Morchella esculenta, Mes*-19, and *Mes*-6 belongs to Esculenta clade (Figures 3A–C).”

The corrected sentence appears below:

“A total of 101 individuals clustered with 10 phylogenetic species, including *Mel*-14, *M. deliciosa*/*Mel*-13, *M. norvegiensis* = *M. eohespera*/*Mel*-19, *Morchella eximia*/*Mel*-5, *Morchella costata, Morchella sextelata*/*Mel*-6, *Morchella septimelata*/*Mel*-7, *Morchella purpurascens*/*Mel*-20, *Mel*-33, and *Morchella pulchella*/*Mel*-31 belongs to Elata clade (Figures 2A–C); and a total of 101 individuals clustered with 8 phylogenetic species, including *M. vulgaris* = *M. spongiola*/*Mes*-5, *Mes*-9, *Mes*-12, *Mes*-26, *Morchella crassipes, Morchella esculenta*/*Mes*-8, *Mes*-19, and *Mes*-6 belongs to Esculenta clade (Figures 3A–C).”

A correction has been made to the **Results** section, paragraph 2. This sentence previously stated:

“The phylospecies of *M. eohespera, Mel*-13, *Mel*-14, *M. eximia, M. costata, M. esculenta, M. crassipes*, and….”

The corrected sentence appears below:

“The phylospecies of *M. norvegiensis* = *M. eohespera*/*Mel*-19, *M. deliciosa*/*Mel*-13, *Mel*-14, *Morchella eximia, M. costata, M. esculenta*/*Mes*-8, *M. crassipes*, and….”

A correction has been made to the **Results** section, paragraph 3. This sentence previously stated:

“such as the *Mel*-13, *Mel*-14, *M. eohespera*, suggests that not all of the *Morchella* species were narrowly distributed;”

The corrected sentence appears below:

“such as the *M. deliciosa*/*Mel*-13, *Mel*-14, *M. norvegiensis* = *M. eohespera*/*Mel*-19, suggests that not all of the *Morchella* species were narrowly distributed;”

A correction has been made to the **Results** section, paragraph 5. This sentence previously stated:

“(1) *M. spongiola*, there were…; (2) *M. esculenta* was widely…”

The corrected sentence appears below:

“(1) *M. vulgaris* = *M. spongiola*/*Mes*-5 there were…; (2) *M. esculenta*/*Mes*-8 was widely…”

A correction has been made to the **Results** section, paragraph 6. This sentence previously stated:

“(4) *M. eohespera* (Elata clade), were distributed in…; (5) *Mel*-13 and *Mel*-14 are widely distributed in Eurasia, especially in the QTPs. *Mel*-13 and…”

The corrected sentence appears below:

“(4) *M. norvegiensis* = *M. eohespera*/*Mel*-19 (Elata clade), were distributed in…; (5) *M. deliciosa*/*Mel*-13 and *Mel*-14 are widely distributed in Eurasia, especially in the QTPs. *M. deliciosa*/*Mel*-13 and…”

A correction has been made to the **Discussion** section, paragraph 3. This sentence previously stated:

“In our data, *M. eohespera* was differentiated at 50.24 Mya with the new uplift belts of Tengchong-Bango formatted and the uplift area of Songpan-Ganzi shrank to the east during Eocene; *M. eohespera* in the middle latitudes region were differentiated at 34.24 Mya with the further uplifted of Kunlun-Algin-Qilian during the Oligocene; ”

The corrected sentence appears below:

“In our data, *M. norvegiensis* = *M. eohespera*/*Mel*-19 was differentiated at 50.24 Mya with the new uplift belts of Tengchong-Bango formatted and the uplift area of Songpan-Ganzi shrank to the east during Eocene; *M. norvegiensis*=*M. eohespera*/*Mel*-19 in the middle latitudes region were differentiated at 34.24 Mya with the further uplifted of Kunlun-Algin-Qilian during the Oligocene;”

A correction has been made to the **Discussion** section paragraph 4. This sentence previously stated:

“(ii) provincialism in the QTPs: the specific local distributions of two species in the Elata clade (*M. pulchella*/*Mel*-12, *M. purpurascens*/*Mel*-20) were unique (only in the Tibet region).”

The corrected sentence appears below:

“(ii) provincialism in the QTPs: the specific local distributions of two species in the Elata clade (*M. pulchella*/*Mel*-31, *M. purpurascens*/*Mel*-20) were unique (only in the Tibet region).”

A correction has been made to the **Discussion** section, paragraph 6. This sentence previously stated:

“For example, in *M. eohespera*, the divergence time was estimated at 52.25 Mya, which was earlier than that of Europe (Supplementary Figure S4).”

The corrected sentence appears below:

“For example, in *M. norvegiensis* = *M. eohespera*/*Mel*-19, the divergence time was estimated at 52.25 Mya, which was earlier than that of Europe (Supplementary Figure S4).”

The authors apologize for these errors and state that this does not change the scientific conclusions of the article in any way. The original article has been updated.

