# Supplementary material for: Out of the Qinghai-Tibetan plateau: Origin, evolution and historical biogeography of Morchella (both Elata and Esculenta clades)
Source: Front Microbiol. 2022 Dec 28;13:1078663. doi: 10.3389/fmicb.2022.1078663 (PMC9832445; doi:10.3389/fmicb.2022.1078663)
Supplement: Supplementary file 2 [file Data_Sheet_1.docx]

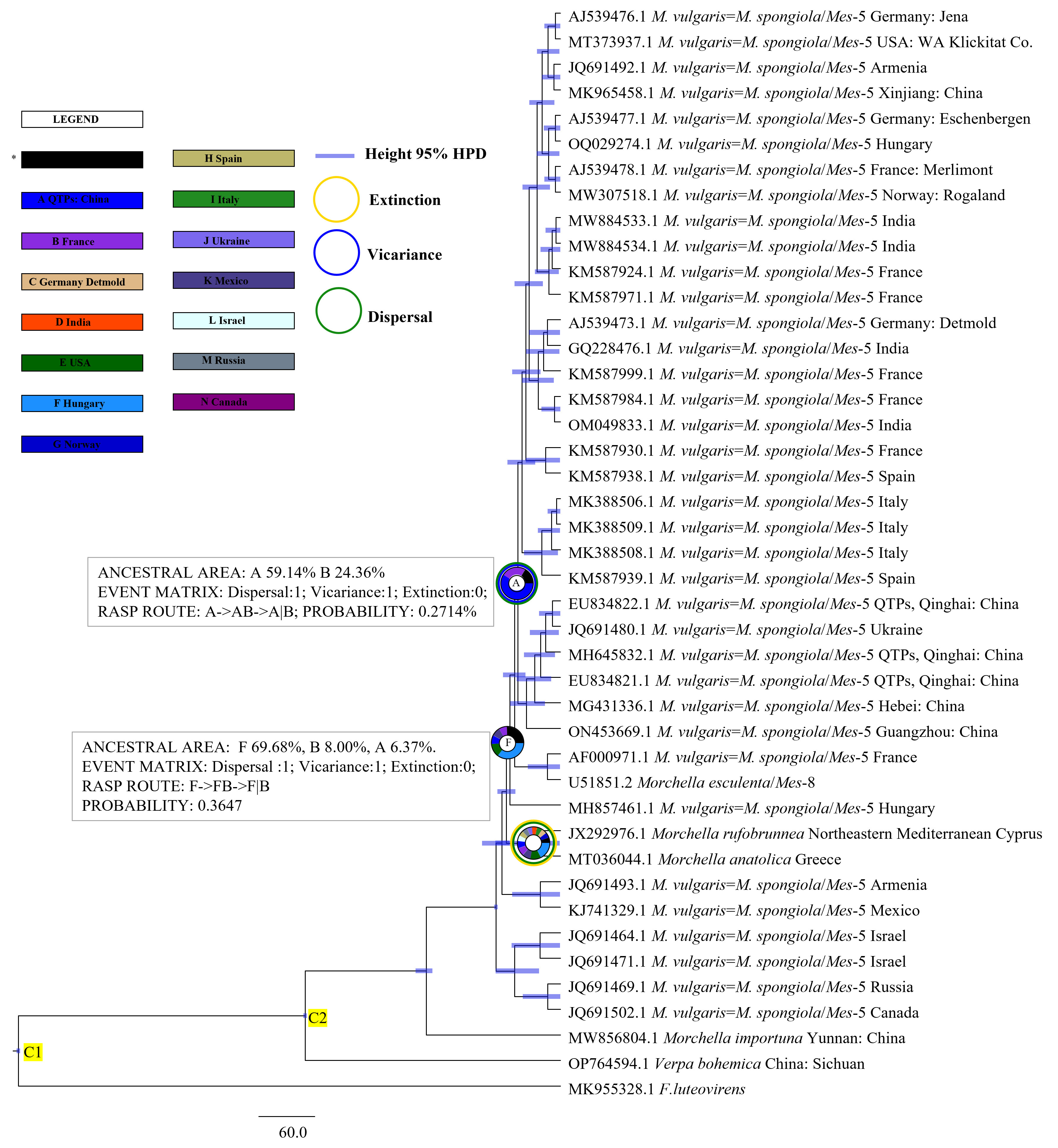


Fig.S1 Differentiation time estimation and ancestral region reconstruction in *Morchella spongiola*. The divergence times were generated by molecular clock analysis using the ITS dataset. The chronogram was obtained using the Ascomycota-Basidiomycota divergence time of 582.08 Mya as the C1. The *Morchella*-*Verpa bohenica* divergence time of 274.06 Mya as the C2. The geological time scale is millions of years ago (Mya). The pie chart in each node indicates the possible ancestral distributions inferred from Bayesian Binary MCMC analysis (BBM) implemented in RASP, including QTPs (A), Europe: Germany(B), Europe: Germany (B), Asia: India (D), Europe: Ukraine (E), North America (F), China: Hebei (G), China: Guangzhou (H). The yellow circle around the pie charts indicates possible extinction events as suggested by BBM analysis.

**
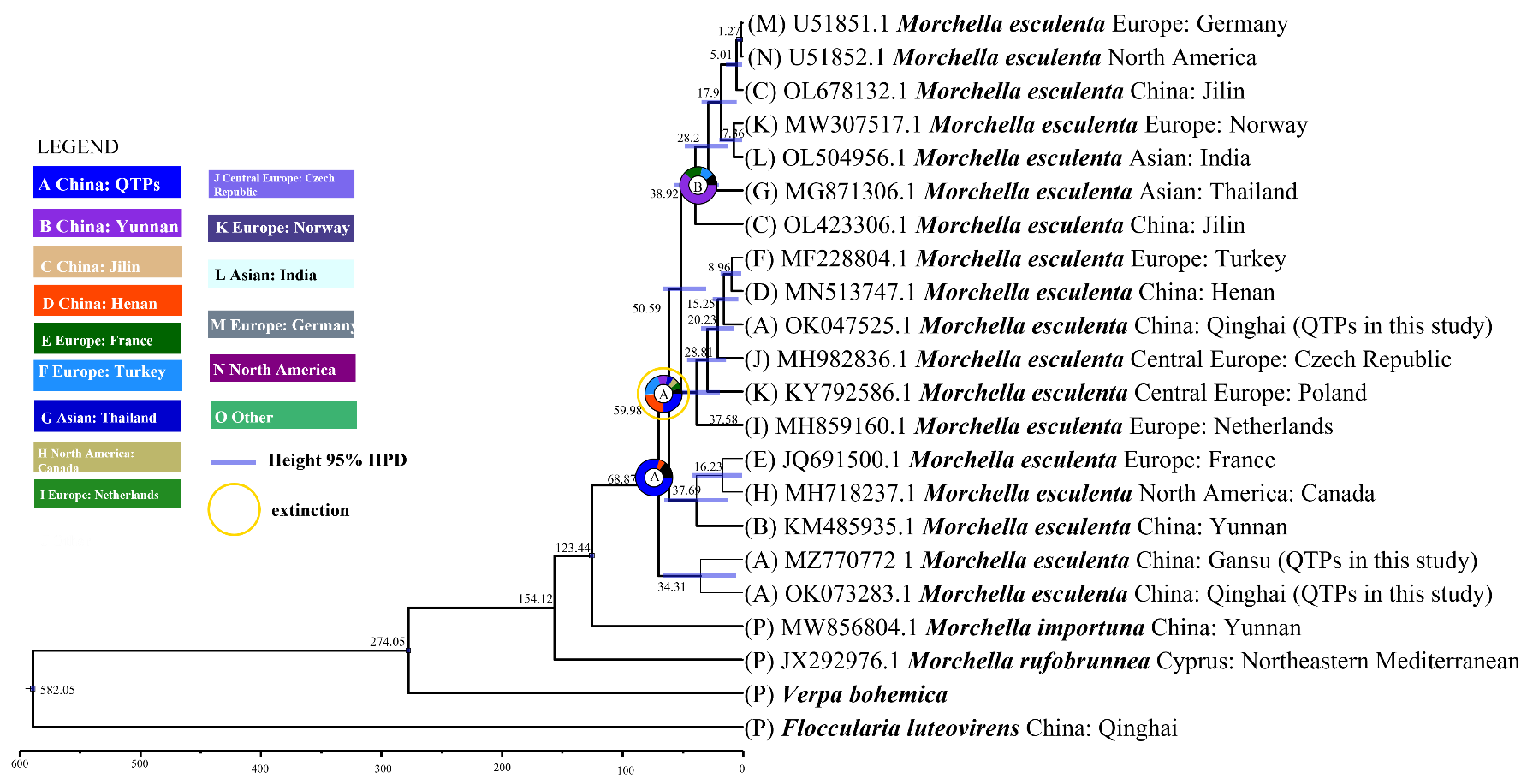
**

**Fig.S2 Differentiation time estimation and ancestral region reconstruction in *Morchella esculenta*.** The divergence times were generated by molecular clock analysis using the ITS dataset. The chronogram was obtained using the Ascomycota-Basidiomycota divergence time of 582.08 Mya as the C1. The *Morchella*-*Verpa bohenica* divergence time of 274.06 Mya as the C2. The geological time scale is millions of years ago (Mya). The pie chart in each node indicates the possible ancestral distributions inferred from Bayesian Binary MCMC analysis (BBM) implemented in RASP, including QTPs (A), China: Yunnan (B), China: Jilin (D), China: Henan (D), Europe: France (E), Europe: Turkey (F), Asia: Thailand (G), Asia: India (H), Europe: Netherlands (I), Central Europe: Czech Republic (J), Central Europe: Poland (K), Asia: India (L), Europe: Germany (M), North America (N). The yellow circle around the pie charts indicates possible extinction events, the blue circle indicates possible vicariance events as suggested by BBM analysis.

**
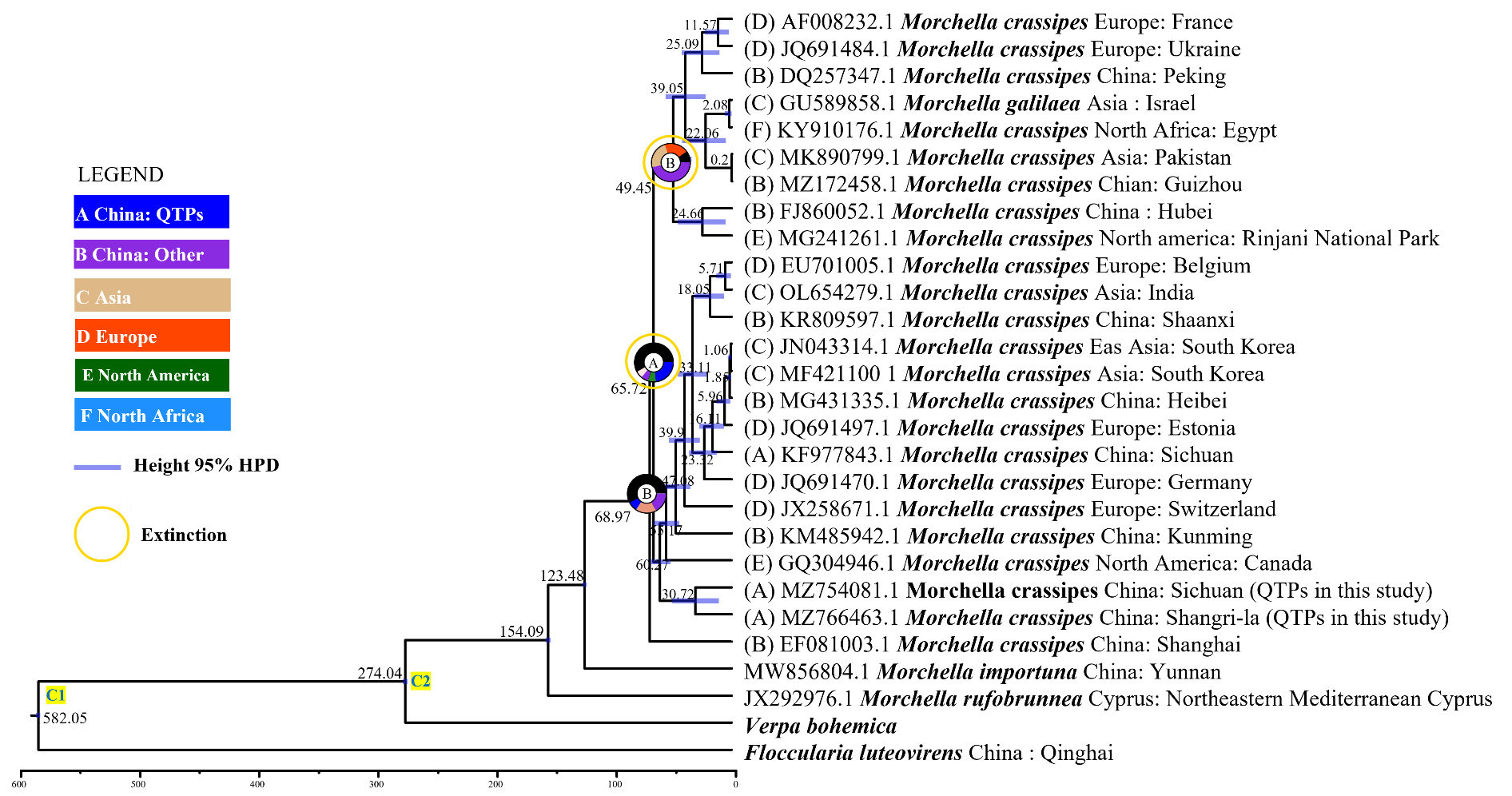
**

**Fig.S3 Differentiation time estimation and ancestral region reconstruction in *Morchella crassipes.*** The divergence times were generated by molecular clock analysis using the ITS dataset. The chronogram was obtained using the Ascomycota-Basidiomycota divergence time of 582.08 Mya as the C1. The *Morchella*-*Verpa bohenica* divergence time of 274.06 Mya as the C2. The geological time scale is millions of years ago (Mya). The pie chart in each node indicates the possible ancestral distributions inferred from Bayesian Binary MCMC analysis (BBM) implemented in RASP, including QTPs (A), China: Yunnan, Peking, Guizhou, Hubei, Shanghai, Heibei, Shaanxi, Sichuan (B), Asia: South Korea (C), Europe: France, Ukraine, Belgium, Estonia, Germany, Switzerland (D), North America: Rinjani National Park, Canada (E), and North Africa: Egypt (F).

**Fig.S4 Differentiation time estimation and ancestral region reconstruction in *Morchella eohespera.*** The divergence times were generated by molecular clock analysis using the ITS dataset. The chronogram was obtained using the Ascomycota-Basidiomycota divergence time of 582.08 Mya as the C1. The *Morchella*-*Verpa bohenica* divergence time of 274.06 Mya as the C2. The geological time scale is millions of years ago (Mya). The pie chart in each node indicates the possible ancestral distributions inferred from Bayesian Binary MCMC analysis (BBM) implemented in RASP, including QTPs (A), North America: British Columbia, Canada, Alaska, Skamania (B), and Central Europe: Czech Republic (C). The yellow circle around the pie charts indicates possible extinction events as suggested by BBM analysis.

**Fig.S5 Differentiation time estimation and ancestral region reconstruction in *Mel*-13 and *Mel*-14.** The divergence times were generated by molecular clock analysis using the ITS dataset. The chronogram was obtained using the Ascomycota-Basidiomycota divergence time of 582.08 Mya as the C1. The *Morchella*-*Verpa bohenica* divergence time of 274.06 Mya as the C2. The geological time scale is millions of years ago (Mya). The pie chart in each node indicates the possible ancestral distributions inferred from Bayesian Binary MCMC analysis (BBM) implemented in RASP, including China: Qinghai (A), China: Gansu (B), China: Tibet (C), and Europe: Turkey(D).

**
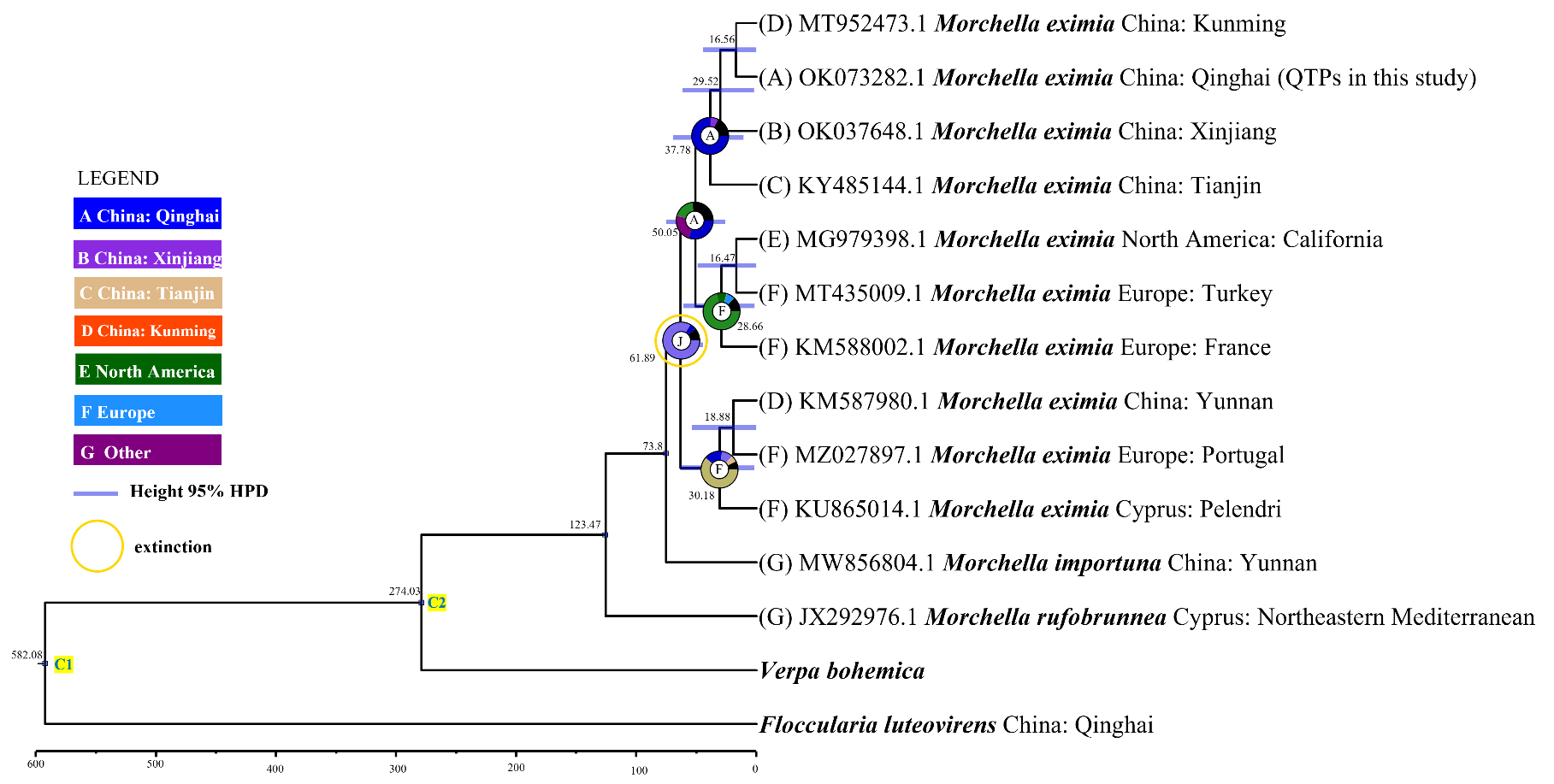
**

**Fig.S6 Differentiation time estimation and ancestral region reconstruction in *Morchella eximia*.** The divergence times were generated by molecular clock analysis using the ITS dataset. The chronogram was obtained using the Ascomycota-Basidiomycota divergence time of 582.08 Mya as the C1. The *Morchella*-*Verpa bohenica* divergence time of 274.06 Mya as the C2. The geological time scale is millions of years ago (Mya). The pie chart in each node indicates the possible ancestral distributions inferred from Bayesian Binary MCMC analysis (BBM) implemented in RASP, including China: Qinghai (A), China: Xinjiang (B), China: Tianjin (C), China: Yunnan (D), North America: California (E), Europe: Turkey, France, Portugal, Pelender (F).

**
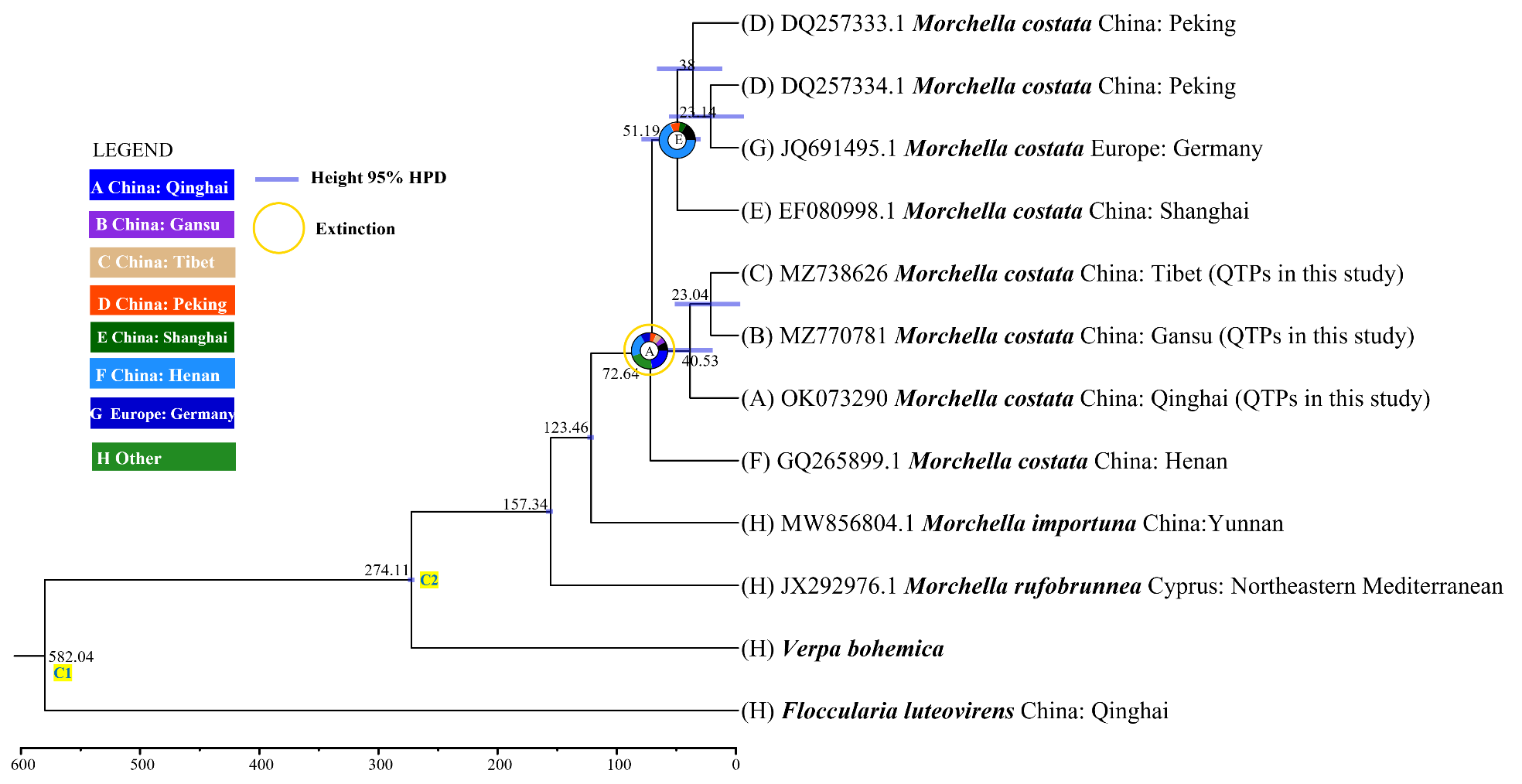
**

**Fig.S7 Differentiation time estimation and ancestral region reconstruction in *Morchella costata.*** The divergence times were generated by molecular clock analysis using the ITS dataset. The chronogram was obtained using the Ascomycota-Basidiomycota divergence time of 582.08 Mya as the C1. The *Morchella*-*Verpa bohenica* divergence time of 274.06 Mya as the C2. The geological time scale is millions of years ago (Mya). The pie chart in each node indicates the possible ancestral distributions inferred from Bayesian Binary MCMC analysis (BBM) implemented in RASP, including China: Qinghai (A), China: Gansu (B), China: Tibet (C), China: Peking (D), China: Shanghai (E), China: Henan (F), Europe: Germany (G).
